# Effect of Early Calf-Hood Nutrition on the Transcriptional Regulation of the Hypothalamic-Pituitary-Testicular axis in Holstein-Friesian Bull Calves

**DOI:** 10.1038/s41598-018-34611-4

**Published:** 2018-11-08

**Authors:** A. M. English, C. J. Byrne, P Cormican, S. M. Waters, S. Fair, D. A. Kenny

**Affiliations:** 1 Animal and Bioscience Research Department, Teagasc Grange, Dunsany C15 PW93 Co. Meath, Ireland; 2 Laboratory of Animal Reproduction, Department of Biological Sciences, University of Limerick, V94 T9PX Limerick, Ireland; 3 School of Agriculture and Food Science, University College Dublin, Belfield D04 N2E5 Dublin, Ireland

## Abstract

The aim of this study was to investigate the effect of early calf-hood nutrition on the transcriptomic profile of the arcuate nucleus of the hypothalamus, anterior pituitary and testes in Holstein-Friesian bulls. Holstein-Friesian bull calves with a mean (±S.D.) age and bodyweight of 19 (±8.2) days and 47.5 (±5.3) kg, respectively, were offered a high (n = 10) or low (n = 10) plane of nutrition in order to achieve an overall growth rate of 1.2 and 0.5 kg/day. At 126 (±3) days of age, calves were euthanized, hypothalamus (arcuate region), anterior pituitary and testicular parenchyma samples were harvested and RNAseq analysis was performed. There were 0, 49 and 1,346 genes differentially expressed in the arcuate nucleus, anterior pituitary and testicular tissue of bull calves on the low relative to the high plane of nutrition, respectively (P < 0.05; False Discovery Rate <0.05). Cell cycle processes in the anterior pituitary were down regulated in the low relative to the high plane of nutrition; there was no differential expression of genes related to reproductive processes. Gene expression involved in cholesterol and androgen biosynthesis in the testes were down regulated in animals on the low plane of nutrition. This study provides insight into the effect of early life plane of nutrition on the regulation of the HPT axis.

## Introduction

Dairy bulls are now selected as potential artificial insemination (AI) sires soon after birth using genomic selection^1^. There is increasing evidence for a positive effect of the plane of nutrition during calfhood on the early onset of puberty in the bull^2–4^. It is desirable not only for such bulls to reach puberty earlier but also that they have the capability of producing high volumes of good quality semen early in life, particularly within the context of seasonal dairy production systems. It is critically important, therefore, to gain a better understanding of the effect of early calf-hood nutrition on the biochemical pathways and key factors affecting sperm production to facilitate the design of improved rearing protocols for such genetically elite and valuable bulls.

Enhanced nutrition in the early calf-hood period has been shown to positively impact the hypothalamic gonadotropin releasing hormone (GnRH) pulse generator and its action on the anterior pituitary gland; thereby advancing the age at onset of puberty in bulls^5^. The hypothalamus is widely acknowledged as the homeostatic regulator of the body^6^. Metabolic signals are sent from organs such as the liver (IGF-1), pancreas (insulin) and adipose tissue (leptin, adiponectin) and received by metabolic sensing neurons involved in satiety and energy homeostasis within the arcuate (ARC) nucleus. Such biochemical messages are mediated by proteins including neuropeptide Y (NPY), agouti-related protein (AgRP)^7,8^ as well as kisspeptin (Kiss)^9,10^. These metabolic sensing neurons stimulate GnRH release thereby affecting reproductive function.

The anterior pituitary is the principal regulator for growth, metabolism and reproduction via the synthesis and/or release of an array of hormones that control these functions in multiple peripheral organs^11^. The gonadotrophic cells in the anterior pituitary are characterised by the expression of GnRH receptors; which are responsible for the regulation of testicular function through secretion of gonadotropins, luteinizing hormone (LH) and follicle stimulating hormone (FSH)^12^. The age at which bulls of dairy breeds attain puberty can range from 8–11 months^13^, although this is influenced by management. There is a transient rise of LH which occurs between 8 and 20 weeks of age, with a peak at 12–15 weeks, declines between 20 and 24 weeks of age^14^. Restricted nutrition during calfhood has been reported to affect steroidogenesis in the testes via inhibition of the magnitude of the hypothalamic GnRH pulse and therefore, the response of the anterior pituitary^5^.

The effect of level of nutrition offered to calves’ during the first six months of life on the early gonadotropin rise and the age at which puberty is reached cannot be rectified by the level of nutrition received thereafter^2,4,5^. We have recently demonstrated that Holstein-Friesian bulls fed a high plane of nutrition for the first six months of life reached puberty approximately one month earlier than bulls on a lower plane irrespective of their plane of nutrition during the subsequent months^2^.

Studies have been carried out in heifers using microarray, *in situ* hybridisation and immunohistochemical technologies to investigate the effect of early life nutrition on the molecular control of the hypothalamus and its knock on effects on age at onset of puberty but there is a lack of information on the nutritional influence on the molecular control of the HPT axis of the bull calf^15–17^. Recent advances in deep-sequencing technology provides the opportunity for in-depth insight into the global transcriptome of key biologically important tissues. The hypothesis under investigation in this study was that the global transcriptomic profiles of hypothalamic-pituitary-testicular tissues would be affected by plane of nutrition during the early calf-hood period in Holstein-Friesian bulls.

## Material and Methods

All procedures involving animals were approved by the Teagasc Animal Ethics Committee (TAEC30/2013) licensed by the Health Products Regulatory Authority (licence number AE19132/P013) in accordance with the European Union Directive 2010/36/EU.

### Animal Model

This experiment was conducted as part of a larger study designed to examine the effect of early calfhood nutrition on the molecular control of the HPT axis. The animal model and management has previously been described by Byrne *et al*.^18^. Briefly, twenty Holstein-Friesian bull calves with a mean (±S.D.) age and bodyweight of 19 (±8.2) days and 47.5 (±5.3) kg, respectively, were sourced from commercial dairy farms blocked on age, sire, liveweight and farm of origin. After five days acclimatisation, calves were assigned to either high or low plane of nutrition. Calves were individually fed milk replacer and concentrates (Tables 1 and 2) using an electronic feeding system (Forster-Tecknik Vario; Engen, Germany). Calves on the high plane of nutrition received 1200 g of milk replacer reconstituted to 8 L daily, together with concentrate *ad libitum*. Calves on the low plane of nutrition were allocated 500 g of milk replacer reconstituted to 4 L plus a maximum of 1 kg of concentrate daily. Diets were designed based on National Research Council guidelines (NRC 2001). Calves on both treatments were weaned when consuming a minimum of 1 kg of concentrate for three consecutive days, at a mean age of 82 (±3.9) days. There was no difference between the two groups in mean age at weaning. Following weaning, the high plane of nutrition group was offered concentrate feed *ad libitum*, while the low plane of nutrition group was offered 1 kg of concentrate, daily. All calves had daily access to approximately 0.5 kg of straw each and a constant supply of fresh water.

**Table 1:** Chemical composition of milk replacer.

	Milk Replacer
**Chemical composition (g/kg)**	
ADF	12.0 ± 1.98
Crude ash	65.7 ± 2.22
CP	216.3 ± 1.24
DM (%)	96.7 ± 0.15
NDF	5.1 ± 1.00
Oil B	235.0 ± 44.10

**Table 2:** Diet and chemical composition of concentrate diet offered.

	Concentrate
**Diet composition (%)**	
Rolled Barley	26.5
Soya bean meal	25
Maize	15
Beet pulp	12.5
Soya hulls	12.5
Molasses	5
Mineral and vitamins	2.5^a^
Vegetable oil	1
**Chemical composition (g/kg)**	
ADF	103.1 ± 6.76
Crude ash	68.8 ± 0.91
CP	167.9 ± 1.86
DM (%)	88.9 ± 0.66
NDF	204.3 ± 18.2
Oil B	30.8 ± 0.72

^a^Mineral and vitamin composition: vitamin A (10 mIU/kg), vitamin D_3_, (2 mIU/kg), vitamin E (40 mg/kg), iodine (8 mg/kg), cobalt (40 mg/kg), copper (88 mg/kg), manganese (81 mg/kg), zinc (139 mg/kg) and selenium (11 mg/kg). ^b^Mineral and vitamin composition for concentrate post puberty is the same as above with the exception of copper (80 mg/kg). ^c^Oil B = Acid hydrolysis.

### Tissue collection

The calves were euthanized at a mean age of 126 (±1.1) days of age, using an intravenous overdose of sodium pentobarbitone. The timing of slaughter was chosen as all calves at this stage would have been expected to have experienced an endogenous transient LH rise^13^ and the timing and magnitude of this rise affects would have affected testicular testosterone synthesis. Blood samples were collected on a fortnightly basis to determine systemic concentrations of LH and was quantified by RIA as previously described by Byrne *et al*.^18^. Death was confirmed by exsanguination followed by decapitation. The skullcap was opened and the brain was removed from the skull by severing the infundibulum, optic nerves and brain stem. Tissue enclosing the ARC nucleus region of the hypothalamus was dissected according to Komatsu *et al*.^19^. Two small triangular sections containing the arcuate nucleus were taken from either side of the bottom of the third ventricle. The pituitary gland was removed from the sella turcica and anterior and posterior sections of the pituitary gland were separated. The testes were excised and the tunica albuginea, epididymides and any excess connective tissue removed. Two sections of the parenchyma were dissected from the middle region of each testis. All samples were washed in sterile Dulbecco’s phosphate-buffered saline (DPBS), snap-frozen in liquid nitrogen, and subsequently stored at −80 °C pending further processing.

### RNA isolation and purification

Total RNA was extracted from each tissue sample using RNeasy Universal plus Kit (Qiagen, Manchester, UK). The quantity and quality of the RNA isolated was determined using the same procedure as described by English *et al*.^20^. RNA integrity number (RIN) for all samples was greater than 8. Further RNA library preparation, sequencing and analysis were carried out as described by English *et al*.^20^.

Sixty (20 calves × 3 tissues) cDNA libraries were prepared from high quality RNA using an Illumina TruSeq RNA Sample Preparation kit v2 following the manufacturer’s instructions (Illumina, San Diego, CA, USA).

Following sequencing two testes samples from the low plane of nutrition and one anterior pituitary sample from the high plane of nutrition were removed from the study due to low sequence reads.

## Results

### Animal performance

Weight gain and other indicators of animal performance were outlined previously^21^. In brief, calves on a high plane of nutrition had larger testes, greater seminiferous tubule diameter, more mature spermatogenic cells and more Sertoli cells (Supplementary Table 4). The effect of a high plane of nutrition on the testicular development was validated at a transcriptional level in this study. There was no effect of nutritional treatment on FSH, LH or testosterone (P > 0.05) but there was an effect of week of sampling on all three hormones (P < 0.0001; Supplementary Fig. 1).

### Differential gene expression

**Figure 1:**
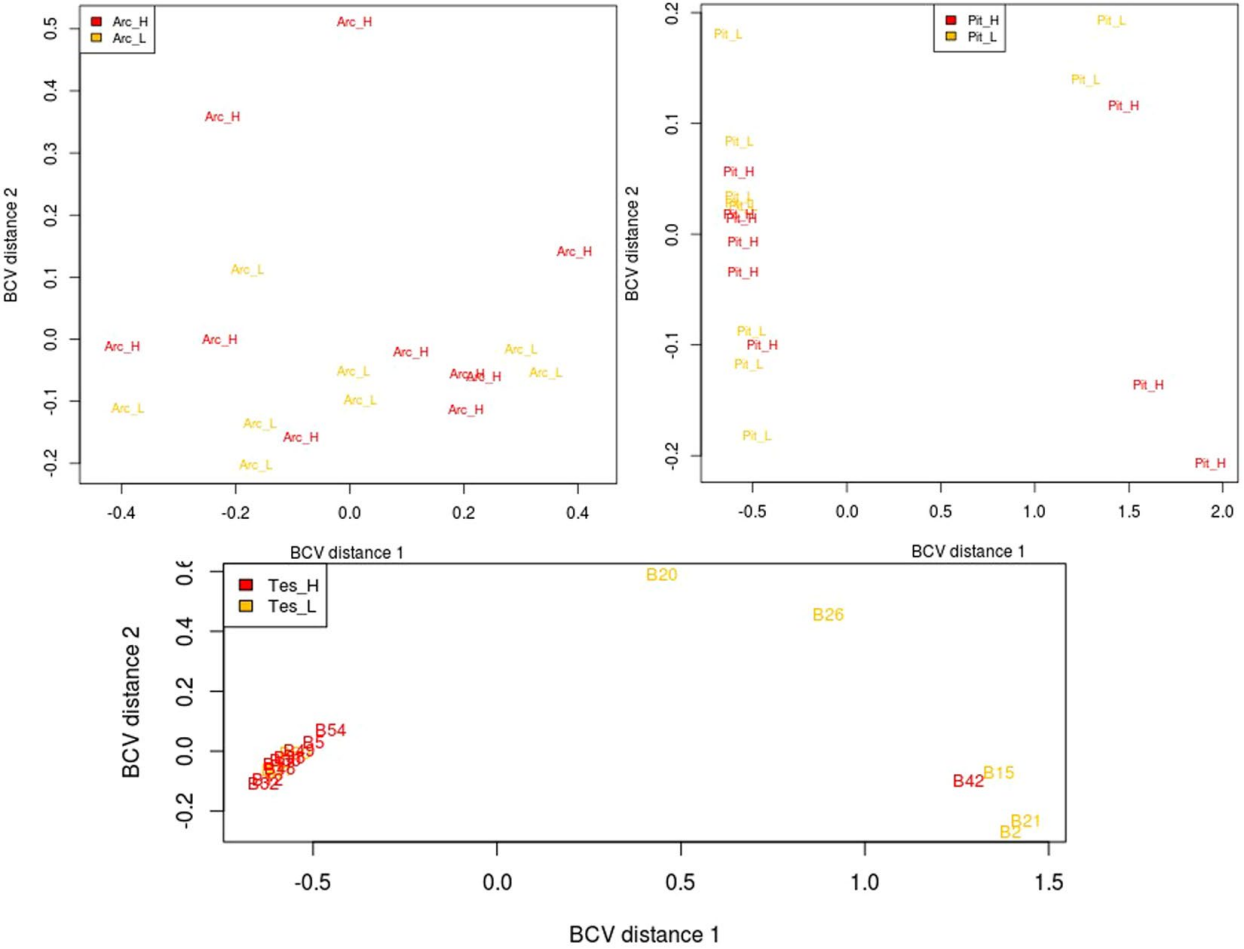
Multidimensional scaling plot which shows the measured similarity of the samples in 2-dimensions. The samples labelled in yellow are the Holstein-Friesian dairy bulls fed on a low plane of nutrition and slaughtered at 18 weeks of age and those labelled in red are the Holstein-Friesian dairy bulls fed on a high plane of nutrition and slaughtered at 18 weeks of age. Arc = Arcuate nucleus, Pit = Anterior Pituitary, Tes = Testes.

The RNAseq data have been deposited in NCBI’s Gene Expression Omnibus and are accessible via GEO series accession number GSE97673. The average number of raw reads for arcuate, anterior pituitary and testes samples were 16.3 million (mean ± SD; 16,319,326 ± 7,160,418), 16.3 million (16,346,430 ± 5,094,647) and 18.3 million (18,324,101 ± 7,135,544), respectively. There were 0, 49 and 1,346 DEG identified in ARC, anterior pituitary and testes tissues between animals on the two divergent planes of nutrition (P < 0.05; False Discovery Rate < 0.05), respectively. A multi-dimensional scaling (MDS) plot was created in Edge-R; the plot showed a lack of separation between samples from the two planes of nutrition with regard to the arcuate nucleus and anterior pituitary tissue but generally good separation between samples from animals on the high compared with the low planes of nutrition in the testes tissue (Fig. 1).

### Pathway analysis

**Figure 2:**
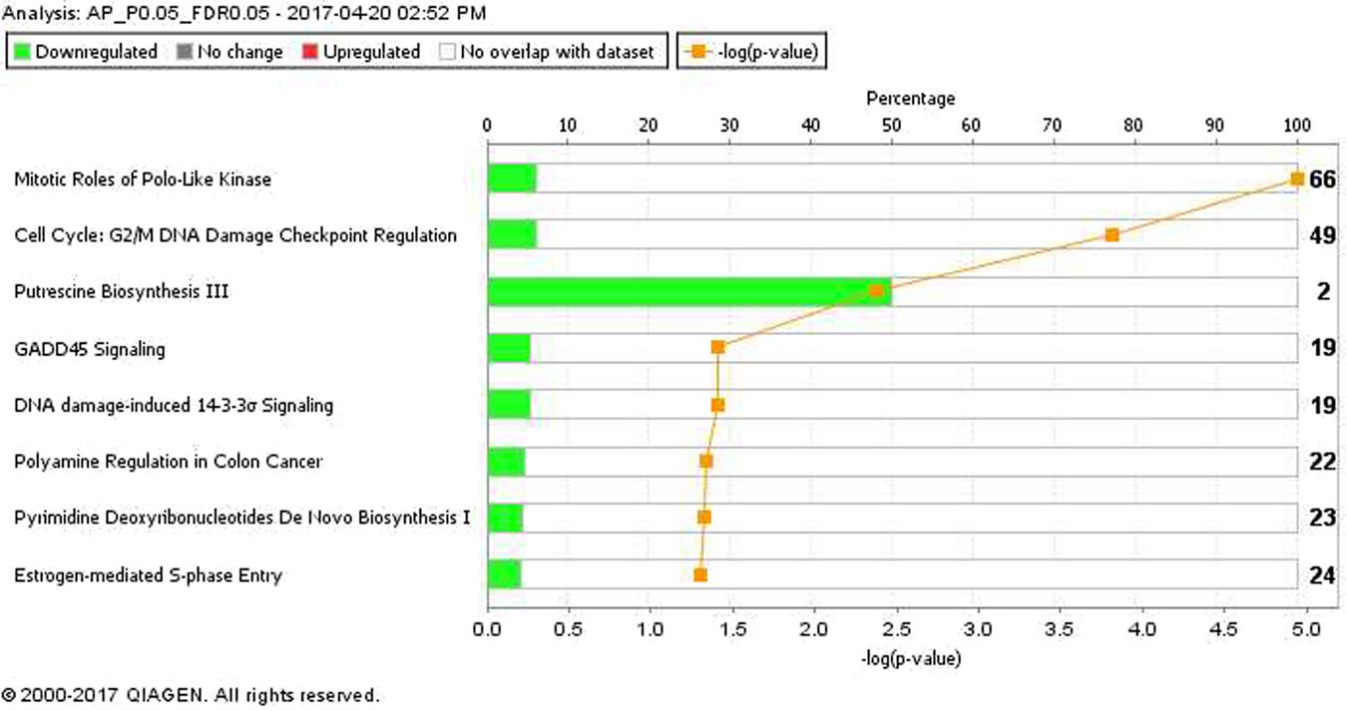
Biochemical pathways statistically significantly enriched in the anterior pituitary of Holstein-Friesian bull calves offered either a low or a high plane of nutrition and, slaughtered at 18 weeks of age. Green and red bars represent genes down and up regulated, respectively, as a percentage of the overall number of genes in each pathway. The significance of each pathway is represented by the yellow line describing −log(p-value). The P-value was calculated by the number of genes from our dataset of differentially expressed genes represented in a particular pathway, divided by the total number of genes in the Canonical Pathway in IPA analysis. Data were analyzed through the use of IPA (QIAGEN Inc., https://www.qiagenbioinformatics.com/products/ingenuitypathway-analysis)^51^.

**Figure 3:**
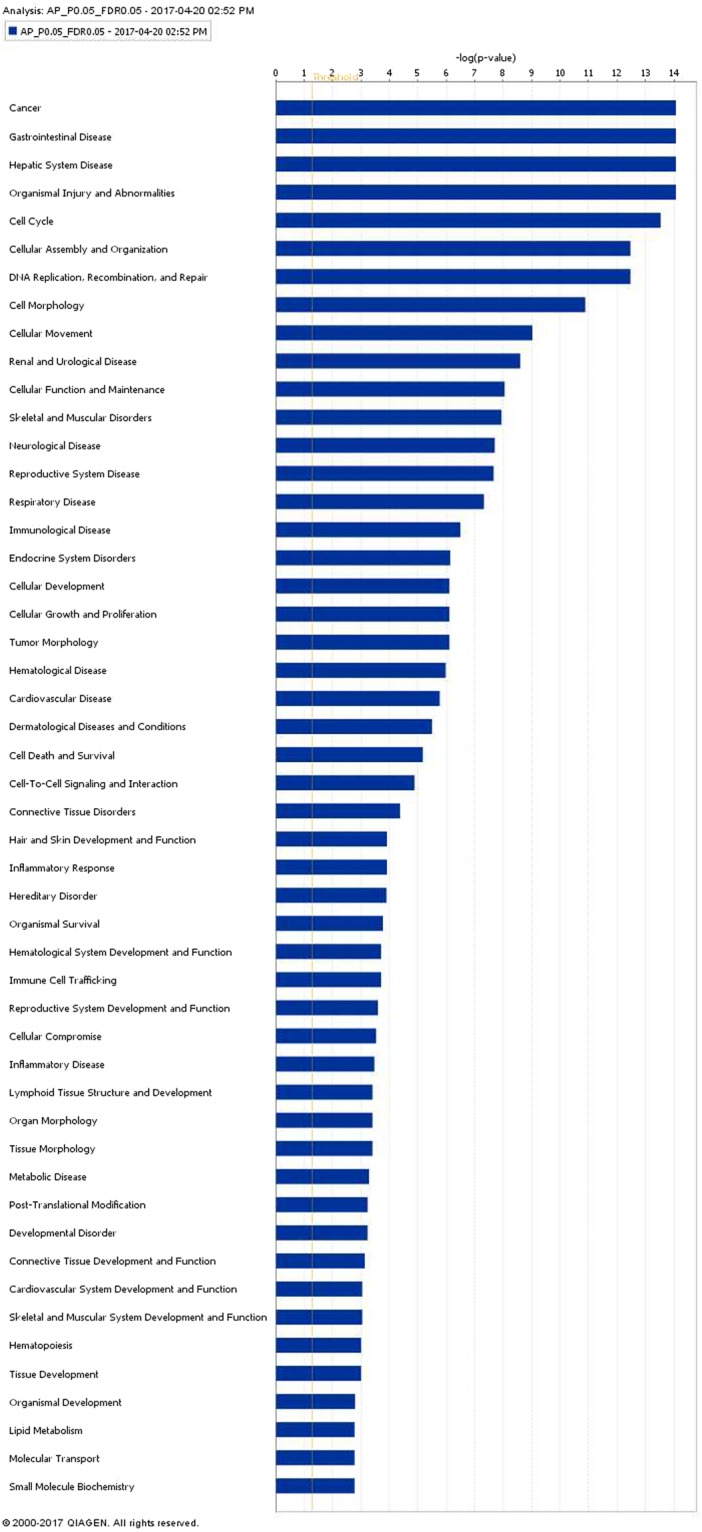
Molecular and cellular function of differentially expressed genes in anterior pituitary tissue of Holstein-Friesian bull calves offered either a low or a high plane of nutrition and and slaughtered at 18 weeks of age. The bars indicate the likelihood [−log (P-value)] that the specific molecular and cellular function was affected by a high plane of nutrition. The threshold line in the bar chart represents a p-value of 0.05. Data were analyzed through the use of IPA (QIAGEN Inc., https://www.qiagenbioinformatics.com/products/ingenuitypathway-analysis)^51^.

**Figure 4:**
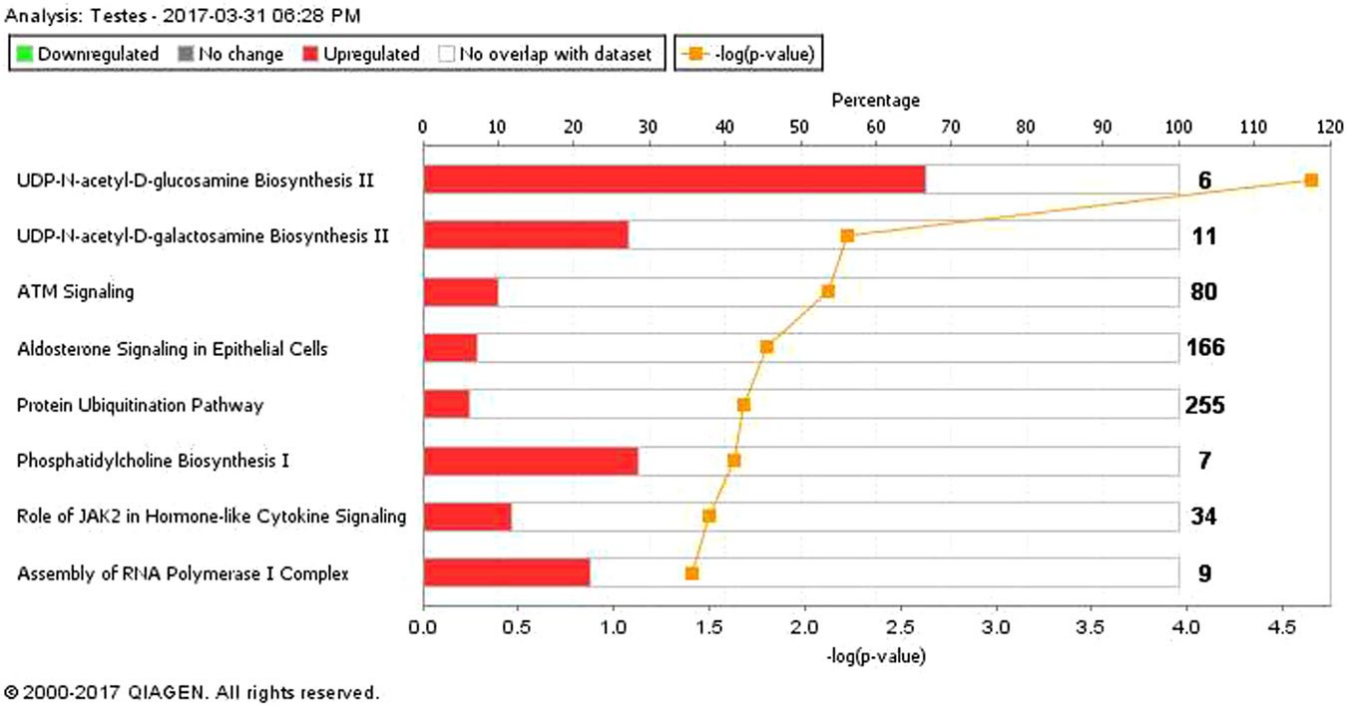
Biochemical pathways statistically significantly enriched in the testes of Holstein-Friesian bull calves offered either a fed on low or a high plane of nutrition and, slaughtered at 18 weeks of age. Green and red bars represent genes down and up regulated, respectively, as a percentage of the overall number of genes in each pathway. The significance of each pathway is represented by the yellow line describing −log(p-value). The P-value is calculated by the number of genes from our dataset of differentially expressed genes represented in a particular pathway, divided by the total number of genes in the Canonical Pathway in IPA analysis. Data were analyzed through the use of IPA (QIAGEN Inc., https://www.qiagenbioinformatics.com/products/ingenuitypathway-analysis)^51^.

**Figure 5:**
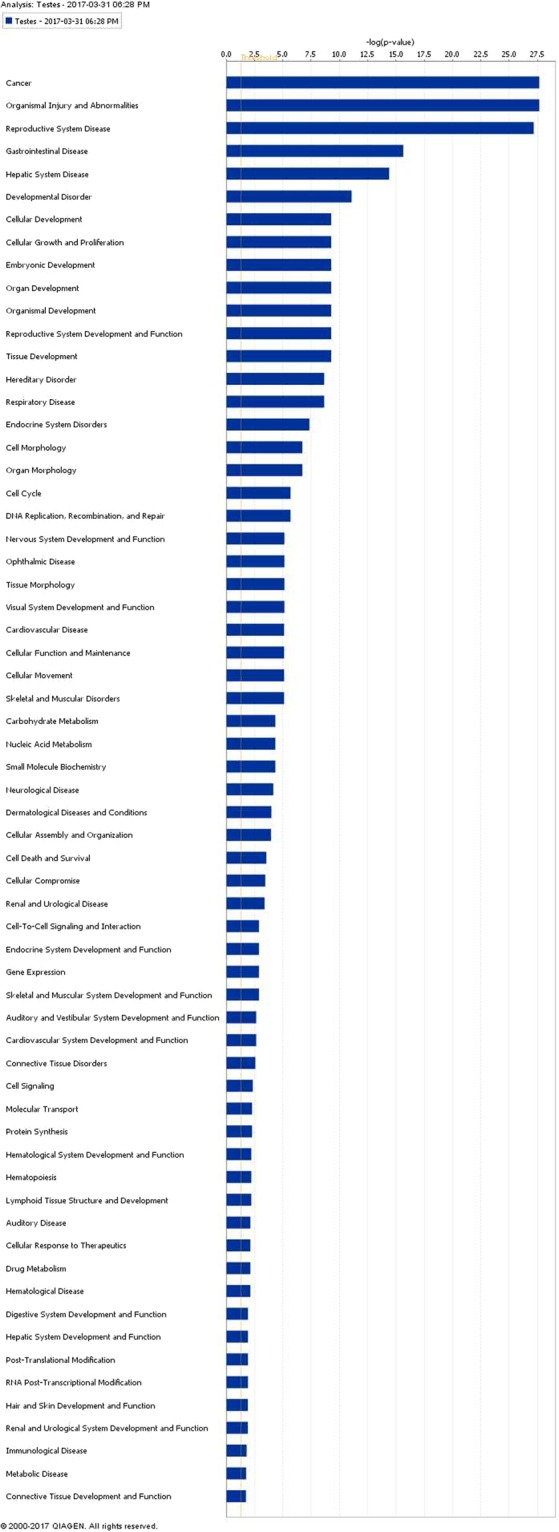
Molecular and cellular function of differentially expressed genes of the testes of Holstein-Friesian bull calves offered either a fed on low or a high plane of nutrition and and slaughtered at 18 weeks of age. The bars indicate the likelihood [−log (P-value)] that the specific molecular and cellular function was affected by a high plane of nutrition. The threshold line in the bar chart represents a p-value of 0.05. Data were analyzed through the use of IPA (QIAGEN Inc., https://www.qiagenbioinformatics.com/products/ingenuitypathway-analysis)^51–54^.

There were 45 DEG out of the original 49 anterior pituitary genes (seven genes with increased expression and 38 genes with decreased expression in the low compared to the high plane of nutrition groups; Supplementary Table 1) and 1,315 DEGs out of the original 1,346 testes genes (1,049 genes with increased expression and 266 genes with decreased expression in the low compared to the high plane of nutrition; Supplementary Table 2) that mapped successfully to a molecular/biological pathway using IPA. Mapped DEG were analysed and allocated to a biological function within IPA. The pathways and processes most affected within anterior pituitary tissue of the calves on the low, compared to the high plane of nutrition were all related to cycle cell processes such as mitotic roles of polo-like kinase (P < 0.0001) and cell cycle: G2/M DNA damage checkpoint regulation (P < 0.0001). Those pathways and processes identified as most differentially expressed within testicular tissue were super pathway of cholesterol biosynthesis (P < 0.0001) and androgen biosynthesis (P < 0.0001). Information on the effect of plane of nutrition on the molecular and cellular functions and on the biochemical pathways of the anterior pituitary and testes are presented in Figs 2–5, respectively. Gametogenesis was predicted to be increased (z-score: 2.279) in testicular tissue of the low compared to the high plane of nutrition calves (Supplementary Table 3). Male infertility function was predicted to be decreased (z-score: −2.236) in the low compared to the high plane of nutrition group.

## Discussion

This is the first study to investigate the effect of plane of nutrition during the early calf-hood period on the transcriptomic profile of the HPT axis in young bulls. Despite large differences in animal growth rate, metabolic status and testicular development between the nutritional treatments groups employed in the study, no DEG were detectable within tissue from the arcuate nucleus region of the hypothalamus. Offering calves a low plane of nutrition caused down regulation of processes involved in cell cycle in the anterior pituitary. The high plane of nutrition induced an increase in DEG in the testes, affecting processes involved in hormone production including androgen and cholesterol biosynthesis in comparison to the low plane of nutrition. Surprisingly, the function of ‘gametogenesis’ was predicted to be upregulated in testicular tissue of calves on the low compared with the high plane of nutrition.

### Cholesterol biosynthesis

Cholesterol is the precursor for all steroid hormones and its availability is vital for their optimal production^22^. Cholesterol can be synthesised *de novo* from acetate, cholesterol ester stores in intracellular lipid droplets or the uptake of cholesterol from low density lipoprotein receptors (*LDLR*)^23^. There are five major stages in the process of cholesterol synthesis. Firstly, two acetyl CoA molecules are converted to 3-hydroxy-3-methylglutaryl-CoA (HMG-CoA) and HMG-CoA is converted to mevalonate. Mevalonate is converted to isopentenyl pyrophosphate (IPP), which is then converted to squalene and the final step is the transformation of squalene to cholesterol. Genes encoding for the enzymes catalysing the biosynthesis of cholesterol including mevalonate kinase (*MVK*), phosphomevalonate (*PMVK*), disphosphomevalonate decarboxylase (*MVD*), dimethylallytranstransferase (*FDPS*), lanosterol synthase (*LSS*), D24 sterol reductase (*DHCR24*), D14 sterol reductase (*TM7SF2*), cholesterol D-isomerase (*EBP*) and 7- dehydrocholesterol reductase (*DHCR7*) were all found to be down regulated in the low compared to the high plane of nutrition group in testicular tissue. This is of interest as under normal physiological conditions, *de novo* cholesterol synthesis replenishes cholesterol stores in Leydig cells^22^.

The low plane of nutrition had a negative effect on genes that mediate the transport of cholesterol. Interestingly, the uptake of high density lipoprotein (HDL) cholesteryl esters via binding Apolipoprotein E (HDL-ApoE)^24^, the selective uptake pathway of HDL cholesterol to the testes via SCARB1^25^ and the LDLR pathway^23^ were downregulated in calves on the low plane of nutrition. Liver X receptor (*LXR*) has been reported to be crucial for maintaining cholesterol homeostasis in murine Sertoli cells^26^. In our study plane of nutrition had no effect on *LXR* gene expression; however, the low plane of nutrition did affect its target genes such as Apolipoprotein E (*APOE*; log fold expression: −2.274) which is expressed in both Sertoli cells and germ cells of sexually immature rats and the Leydig cells of sexually mature rats^27^ and Scavenger receptor B1 (*SCARB1*; log fold expression: −1.816) which is expressed in murine Leydig cells^28^. The expression of *LDLR* was down regulated (log fold expression: −1.789) in calves on the low compared to the high plane of nutrition, indicating that this source of cholesterol inhibited testosterone synthesis as testicular Leydig cells under certain conditions obtain plasma lipoprotein-derived cholesterol for steroid synthesis^29^. In an associated study we analysed blood from the animals employed here and found greater serum testosterone concentrations in the calves on the high compared to the low plane of nutrition (Supplementary Fig. 1). Therefore, down regulation of cholesterol biosynthesis will likely negatively affect androgen biosynthesis.

### Androgen biosynthesis

Androgens are crucial for optimal male reproductive function, playing important roles in maintaining spermatogenesis and sexual function. The rate of testosterone and estradiol production is controlled by gonadotropins, LH and FSH^22^. In our study we observed that *FSHR* (follicle stimulating hormone receptor) was down regulated in the high compared to the low plane of nutrition in the testes (log fold expression: −1.021). In the male, FSH stimulates Sertoli cell proliferation, as well as the induction and maintenance of normal spermatogenesis^30^. Expression of *FSHR* mRNA and number of FSH receptors has been found to decrease with testes development as bulls mature^31^. It has been reported that steroidogenic acute regulatory protein (*StAR*) is the primary determinant in the process of steroid synthesis^32^. The expression of *StAR* mRNA has previously been shown to be expressed in bull testes^33^ and it regulates the transport of cholesterol from the outer into the inner mitochondrial membrane. Treating bulls with GnRH agonist caused increased testosterone biosynthesis and StAR protein in the anterior pituitary^34^. In our study, calves offered the low plane of nutrition had a lower expression of *StAR* (log fold change: −1.914) compared to their contemporaries on the high plane of nutrition. The expression of *StAR* has been reported to be stimulated by IGF-1 in fetal and adult mice Leydig cells^35,36^. This is consistent with previous reports from our group showing greater plasma IGF-1 concentrations when the same animals in this study were offered a high compared with a low plane of nutrition (Supplementary Fig. 1).

There are two pathways involved in the conversion of pregnenolone to testosterone in the Leydig cells, Δ4 or Δ5. In the Δ5 pathway, pregnenolone is converted to 17α-hydroxypregnenolone then to dehydroepiandrosterone and finally to testosterone through either androstenediol or androstenedione. Our data show that this pathway was negatively affected by the low plane of nutrition. The conversion of cholesterol to pregnenolone is catalysed by cytochrome P450 side chain cleavage enzyme (P450scc) located in the inner mitochondrial membrane^37^. The transcription of *CYP11A1* gene encoding P450scc regulates the quantity of P450scc and therefore, the steroidogenic function^23^. In the current study, *CYP11A1* was down regulated in the low compared to the high plane of nutrition. Genes encoding for the enzymes catalysing the formation of testosterone including 3-β-hydroxysteroid dehydrogenase (*HSD3B7* and *HSD3B2*) and steroid D-isomerase (*HSD3B2* and *EBP*) were down regulated in the low compared to the high plane of nutrition, which led to decreased production of testosterone. However, reducing dietary nutrient intake did not down regulate all genes related to testosterone production as *17β-HSD*, which codes for the enzyme responsible for the conversion of androstenedione to testosterone, was not altered by plane of nutrition.

### Gametogenesis

There were 11 of 87 genes displaying upregulated expression in the low plane of nutrition animals consistent with a predicted increase in gametogenesis (spermatogenesis). These genes included *SIAH1*, *RNF2*, *MCM9*, *CTCFL*, *FSHR*, *AGFG1*, *DDX25*, *MCM8* and *KDM3A*, which have all been reported to be involved in aiding gametogenesis^38–40^. This was surprising and may be due to the low plane of nutrition calves experiencing a late spurt of gametogenesis. Male infertility function was predicted to be decreased in the low plane of nutrition compared to the high. There were five genes out of 11 genes which had an expression consistent with an increase in male infertility. These genes including *KMT2E (MLL5)*, *SETX*, *GMCL1*, *SIRT1* and *TDRD5*; which have all been reported to be involved in aiding spermatogenesis in adult mice and humans^41–45^. Gonadotropin regulated testicular RNA helicase (GRTH/DDX25) is regulated by gonadotropin in Leydig cells and germ cells and is necessary for completion of spermatogenesis^46^. In Leydig cells, GRTH is a negative regulator of the expression of genes involved in cholesterol synthesis and transfer (SREBP2, HMG-CoA and StAR) and therefore, exhibiting control over androgen synthesis^47^. However, we found that testosterone concentrations were significantly higher for the calves on the high compared with the low plane of nutrition from 10 weeks of age until their slaughter at 18 weeks of age (Supplementary Fig. 1) and the high calves had larger testes^48^, a greater seminiferous tubule diameter, a greater number of more spermatogenic cells and a greater number of Sertoli cells (Supplementary Table 4). Our research group has also reported on the effect of plane of nutrition on systemic concentrations of testosterone in Holstein Friesian bulls calves at 16 and 32 weeks of age, following an exogenous GnRH challenge^2^. Additionally we have shown Holstein Friesian bulls calves offered a high plane of nutrition pre six months to reach puberty approximately 30 days earlier that their counterparts on a lower dietary allowance^2^. Although both functions were predicted to be upregulated in the calves on the low compared to the high treatment, the testes from the high plane of nutrition calves were at a more mature stage with regard to testes weight, seminiferous tubule diameter, stage of spermatogenesis and Sertoli cell number and lumen development at slaughter.

Differences in metabolic function in the ARC using microarray technologies were reported in heifer calves fed on a high concentrate diet to achieve rapid bodyweight gain, compared to contemporaries offered a high forage diet, from three-seven months of age^15^. This study found that key genes involved in satiety such as *NPY* and *AGRP* were down regulated and *POMC* and *α-MSH* were upregulated in the high concentrate heifers. The genes *NPY/AGRP* are well documented in have opposing roles to that of *POMC/α-MSH* in the control of feeding and energy expenditure^8^. It has been reported that *NPY* acts directly on GnRH neurons and that this may also be the case for *AGRP* as melanocortin agonists have direct action on GnRH neurons^49^. A study also carried out by the same research group using *in-situ* hybridisation and immunohistochemical technologies reported that increased rates of growth during the juvenile period in heifers alter the methylation pattern of genomic DNA from the ARC and such alterations may be linked to advanced age at puberty^16^. Our study however, found no DEG within ARC tissue between treatments. However, the studies reported above involved animals of a different sex, age, nutritional treatment and analytical technologies compared to our study which may have contributed to the difference in findings between studies. Our study is the first to date to utilize next generation sequencing technology to examine the effect of early calf hood nutrition on the global transcriptome of HPT tissue in Holstein-Friesian bull calves. The transcriptional profile of the ARC nucleus and anterior pituitary found in this study does not reflect the phenotype, in that the only DEG found in the anterior pituitary related to cell cycle and not specifically to metabolism or reproductive processes, as might be expected. This may be due to the transitory nature of gene transcripts in the brain^50^. However, while we did not detect DEG in either ARC or the anterior pituitary tissues which were specific to reproductive processes, gene expression profiles for testicular tissue were indicative of prior influence of plane of nutrition on the functionality of the hypothalamic-anterior pituitary axis, upon which testicular development depends.

## Electronic supplementary material

Supplementary file
