# Supplementary material for: Effect of Early Calf-Hood Nutrition on the Transcriptional Regulation of the Hypothalamic-Pituitary-Testicular axis in Holstein-Friesian Bull Calves
Source: Sci Rep. 2018 Nov 8;8:16577. doi: 10.1038/s41598-018-34611-4 (PMC6224434; doi:10.1038/s41598-018-34611-4)
Supplement: Supplementary file 1 — Supplementary file [file 41598_2018_34611_MOESM1_ESM.pdf]

Effect of Early Calf-Hood Nutrition on the Transcriptional Regulation of the Hypothalamic-Pituitary-Testicular axis in Holstein-Friesian Bull Calves

A.M. English[1,2], C. J. Byrne [1,3], P. Cormican [1], S.M. Waters[1], S. Fair [2] and D.A. Kenny [1,3,*]

[1] Animal and Bioscience Research Department, Teagasc Grange, Dunsany, Co. Meath, C15 PW93, Ireland

[2] Laboratory of Animal Reproduction, Department of Biological Sciences, University of Limerick, V94 T9PX, Ireland

[3] School of Agriculture and Food Science, University College Dublin, Belfield, Dublin 4, Dublin, D04 N2E5, Ireland

[*]Corresponding author:

E-mail: david.kenny@teagasc.ie (DK)

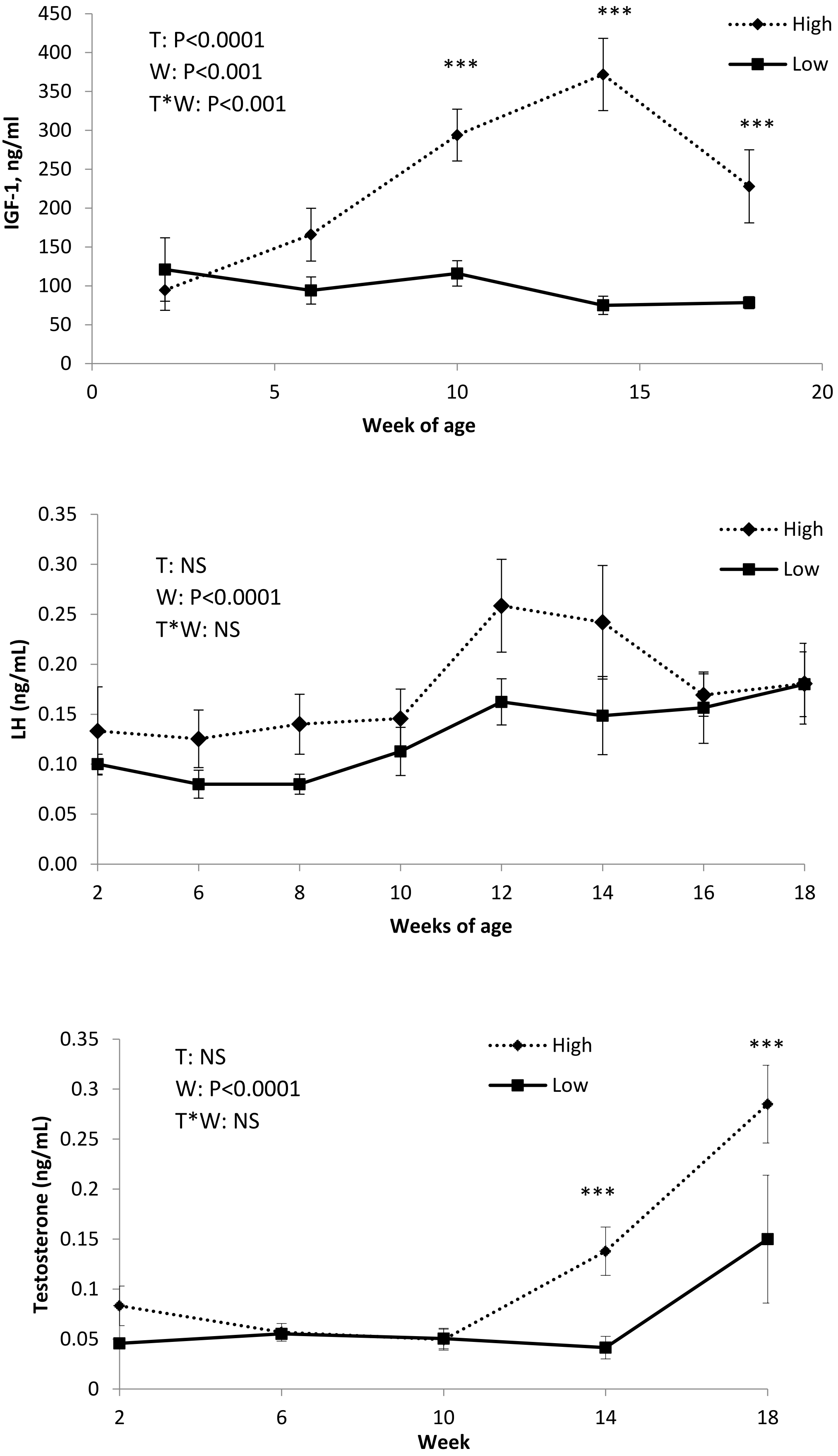
IGF-1, ng/ml
Week of age
T: P<0.0001
W: P<0.001
T*W: P<0.001
High
Low
***
LH (ng/mL)
Weeks of age
T: NS
W: P<0.0001
T*W: NS
High
Low
Testosterone (ng/mL)
Week
T: NS
W: P<0.0001
T*W: NS
High
Low
***

Supplementary Figure 1: Effect of plane of nutrition on the systemic concentrations on IGF-I, LH and Testosterone. Black full line: low plane of nutrition, Black dotted line: high plane of nutrition; T= Treatment, W=Week, T*W= Treatment by Week Interaction, NS= Non significant, *** $P<0.001$, error bars represent standard error of the mean;

Supplementary Table 1: Genes differentially expressed in the anterior pituitary of the Holstein-Friesian dairy bulls fed on low plane of nutrition in comparison to a high plane of nutrition, slaughtered at 18 weeks of age.

Gene Symbol	Gene Name	Expr Log Ratio	Expr P-value	Expr False Discovery Rate (FDR)
ARHGAP11A	Rho GTPase activating protein 11A	-2.382	5.87E-08	4.82E-05
ASPM	abnormal spindle microtubule assembly	-2.122	3.75E-10	7.18E-07
BAZ2A	bromodomain adjacent to zinc finger domain 2A	0.448	1.01E-04	2.90E-02
BIRC5	baculoviral IAP repeat containing 5	-1.86	6.28E-12	2.40E-08
BUB1B	BUB1 mitotic checkpoint serine/threonine kinase B	-1.967	5.74E-10	8.24E-07
C1QTNF6	C1q and tumor necrosis factor related protein 6	-1.442	5.50E-05	1.80E-02
C4orf32	chromosome 4 open reading frame 32	-1.417	1.63E-04	4.04E-02
CASR	calcium sensing receptor	-2.157	7.65E-05	2.26E-02
CD44	CD44 molecule (Indian blood group)	-1.043	9.48E-07	5.44E-04
CDK1	cyclin dependent kinase 1	-1.437	1.41E-06	7.39E-04
CENPE	centromere protein E	-1.596	7.86E-06	3.47E-03
CENPF	centromere protein F	-2.031	2.59E-07	1.66E-04
CKAP2	cytoskeleton associated protein 2	-1.13	1.08E-05	4.43E-03
CKS2	CDC28 protein kinase regulatory subunit 2	-1.502	2.67E-14	1.53E-10
CPXM2	carboxypeptidase X, M14 family member 2	-1.099	7.67E-05	2.26E-02
DAPL1	death associated protein like 1	-1.414	3.92E-05	1.41E-02
DNAH5	dynein axonemal heavy chain 5	0.895	1.64E-05	6.50E-03
DSE	dermatan sulfate epimerase	-0.727	5.18E-05	1.75E-02
ECT2	epithelial cell transforming 2	-1.571	4.34E-06	2.14E-03
HIF3A	hypoxia inducible factor 3 alpha subunit	2.09	7.82E-15	8.98E-11
KCNH8	potassium voltage-gated channel subfamily H member 8	0.666	1.47E-04	3.89E-02
KCNIP1	potassium voltage-gated channel interacting protein 1	1.117	4.75E-05	1.65E-02
KIF11	kinesin family member 11	-2.313	1.00E-07	7.70E-05
KIF23	kinesin family member 23	-1.056	4.48E-06	2.14E-03
KIF20A	kinesin family member 20A	-1.735	5.18E-10	8.24E-07

KIT	KIT proto-oncogene receptor tyrosine kinase	-1.611	4.30E-08	3.80E-05
LCP1	lymphocyte cytosolic protein 1	-0.795	8.36E-06	3.56E-03
LXN	latexin	-0.966	1.19E-04	3.31E-02
MKI67	marker of proliferation Ki-67	-1.829	1.13E-10	3.26E-07
MYH6	myosin heavy chain 6	-2.053	1.21E-04	3.31E-02
NCAPG	non-SMC condensin I complex subunit G	-1.196	3.83E-05	1.41E-02
NUSAP1	nucleolar and spindle associated protein 1	-1.059	6.56E-05	2.09E-02
ODC1	ornithine decarboxylase 1	-0.91	2.36E-07	1.59E-04
PCSK1	proprotein convertase subtilisin/kexin type 1	-0.941	3.87E-05	1.41E-02
PLEKHM3	pleckstrin homology domain containing M3	0.568	1.65E-04	4.04E-02
PRC1	protein regulator of cytokinesis 1	-1.338	4.71E-07	2.85E-04
RAD54L	RAD54-like (S. cerevisiae)	-0.613	2.06E-04	4.93E-02
RRM2	ribonucleotide reductase regulatory subunit M2	-2.436	2.60E-10	5.98E-07
SLC24A2	solute carrier family 24 member 2	1.108	3.47E-08	3.61E-05
SPAG5	sperm associated antigen 5	-1.457	1.44E-07	1.04E-04
SPARC	secreted protein acidic and cysteine rich	-0.672	1.49E-04	3.89E-02
SPON1	spondin 1	-1.113	5.05E-09	5.81E-06
STMN4	stathmin 4	-1.244	1.30E-06	7.10E-04
TOP2A	topoisomerase (DNA) II alpha	-2.277	3.77E-08	3.61E-05
TPX2	TPX2, microtubule nucleation factor	-1.454	1.33E-09	1.70E-06

Supplementary Table 2: Genes differentially expressed in the parenchyma of the testes of the Holstein-Friesian dairy bulls fed on low plane of nutrition in comparison to a high plane of nutrition, slaughtered at 18 weeks of age.

Gene Symbol	Gene Name	Expr Log Ratio	Expr P-value	Expr False Discovery Rate (FDR)
AACS	acetoacetyl-CoA synthetase	-1.26	4.92E-04	1.15E-02
ABCA1	ATP binding cassette subfamily A member 1	0.66	2.45E-03	2.88E-02
ABCA5	ATP binding cassette subfamily A member 5	0.673	1.45E-04	5.95E-03
ABCB6	ATP binding cassette subfamily B member 6 (Langereis blood group)	-1.026	2.38E-05	2.34E-03
ABCC9	ATP binding cassette subfamily C member 9	0.705	4.88E-03	4.26E-02
ABCG2	ATP binding cassette subfamily G member 2 (Junior blood group)	0.746	1.24E-03	1.96E-02
ABHD5	abhydrolase domain containing 5	0.544	4.58E-03	4.10E-02
ABI3BP	ABI family member 3 binding protein	0.881	2.07E-03	2.60E-02
ACADVL	acyl-CoA dehydrogenase, very long chain	-0.688	2.73E-03	3.06E-02
ACE2	angiotensin I converting enzyme 2	0.903	1.81E-04	6.90E-03
ACLY	ATP citrate lyase	-0.839	2.10E-03	2.62E-02
ACSL6	acyl-CoA synthetase long-chain family member 6	0.481	5.05E-04	1.17E-02
ACSS2	acyl-CoA synthetase short-chain family member 2	-1.576	2.76E-05	2.50E-03
ACVR2A	activin A receptor type 2A	0.769	1.70E-03	2.36E-02
ADAD1	adenosine deaminase domain containing 1	1.456	1.12E-05	1.67E-03
ADAM12	ADAM metallopeptidase domain 12	-0.716	5.55E-04	1.23E-02
ADAM23	ADAM metallopeptidase domain 23	0.874	1.28E-03	1.99E-02
ADAM32	ADAM metallopeptidase domain 32	0.782	2.32E-03	2.76E-02
ADAMTS2	ADAM metallopeptidase with thrombospondin type 1 motif 2	-0.951	2.38E-03	2.81E-02
ADAMTS17	ADAM metallopeptidase with thrombospondin type 1 motif 17	-1.076	2.96E-03	3.22E-02
ADAMTSL3	ADAMTS like 3	0.524	6.03E-03	4.83E-02
ADCY3	adenylate cyclase 3	-0.871	7.64E-04	1.49E-02
ADGRG2	adhesion G protein-coupled receptor G2	1.234	2.74E-08	1.07E-04
ADGRG6	adhesion G protein-coupled receptor G6	1.167	1.24E-05	1.78E-03
ADGRV1	adhesion G protein-coupled receptor V1	0.829	1.66E-04	6.55E-03
ADIPOR2	adiponectin receptor 2	-0.76	5.21E-04	1.19E-02
ADNP	activity dependent neuroprotector homeobox	0.489	3.96E-03	3.76E-02

ADSS	adenylosuccinate synthase	0.772	1.86E-03	2.49E-02
AFTPH	aftiphilin	0.666	2.80E-03	3.10E-02
AGBL3	ATP/GTP binding protein like 3	0.804	5.04E-04	1.17E-02
AGFG1	ArfGAP with FG repeats 1	0.503	4.39E-03	4.01E-02
AGFG2	ArfGAP with FG repeats 2	-1.344	1.73E-03	2.38E-02
AGGF1	angiogenic factor with G-patch and FHA domains 1	0.732	9.01E-04	1.65E-02
AGPAT5	1-acylglycerol-3-phosphate O-acyltransferase 5	0.584	4.09E-03	3.84E-02
AHCTF1	AT-hook containing transcription factor 1	0.656	1.99E-03	2.55E-02
AHI1	Abelson helper integration site 1	1.065	3.07E-05	2.60E-03
AHNAK	AHNAK nucleoprotein	0.527	9.06E-04	1.65E-02
AIM1	absent in melanoma 1	0.667	2.02E-03	2.57E-02
AIMP1	aminoacyl tRNA synthetase complex interacting multifunctional protein 1	0.772	1.90E-03	2.50E-02
AK7	adenylate kinase 7	0.627	1.48E-03	2.20E-02
AK9	adenylate kinase 9	1.074	3.68E-04	9.70E-03
AKAP6	A-kinase anchoring protein 6	0.958	1.42E-05	1.94E-03
AKIRIN2	akirin 2	0.531	4.44E-03	4.02E-02
AKT3	AKT serine/threonine kinase 3	0.634	1.79E-03	2.44E-02
ALDH1A1	aldehyde dehydrogenase 1 family member A1	-0.792	6.12E-03	4.87E-02
ALDOC	aldolase, fructose-bisphosphate C	-1.336	5.78E-04	1.25E-02
ALG9	ALG9, alpha-1,2-mannosyltransferase	0.517	2.32E-03	2.76E-02
ALG13	ALG13, UDP-N-acetylglucosaminyltransferase subunit	1.131	1.14E-05	1.67E-03
ALPL	alkaline phosphatase, liver/bone/kidney	-1.436	1.28E-03	1.99E-02
ALS2	ALS2, alsin Rho guanine nucleotide exchange factor	0.76	3.59E-06	9.60E-04
ALS2CR11	amyotrophic lateral sclerosis 2 chromosome region candidate 11	1.459	5.00E-07	5.87E-04
AMIGO1	adhesion molecule with Ig like domain 1	0.539	1.84E-03	2.48E-02
ANGPT4	angiopoietin 4	-1.231	2.84E-05	2.53E-03
ANGPTL2	angiopoietin like 2	-1.442	3.04E-05	2.60E-03
ANGPTL4	angiopoietin like 4	-2.079	2.09E-04	7.37E-03
ANKRD17	ankyrin repeat domain 17	0.531	2.32E-03	2.76E-02
ANKRD26	ankyrin repeat domain 26	0.67	5.99E-03	4.81E-02
ANKRD39	ankyrin repeat domain 39	-0.948	1.48E-03	2.19E-02
ANXA6	annexin A6	-0.639	2.18E-03	2.68E-02

AP1AR	adaptor related protein complex 1 associated regulatory protein	0.807	3.08E-03	3.30E-02
AP2M1	adaptor related protein complex 2 mu 1 subunit	-0.397	1.87E-03	2.49E-02
AP4E1	adaptor related protein complex 4 epsilon 1 subunit	0.767	2.19E-03	2.69E-02
APBB1IP	amyloid beta precursor protein binding family B member 1 interacting protein	0.5	5.55E-03	4.59E-02
APLF	aprataxin and PNKP like factor	0.801	3.58E-03	3.57E-02
APOE	apolipoprotein E	-2.274	2.41E-03	2.84E-02
APPL1	adaptor protein, phosphotyrosine interacting with PH domain and leucine zipper 1	0.578	3.05E-03	3.28E-02
ARFGAP3	ADP ribosylation factor GTPase activating protein 3	-0.548	4.80E-03	4.20E-02
ARFGEF1	ADP ribosylation factor guanine nucleotide exchange factor 1	0.544	5.86E-03	4.72E-02
ARHGAP12	Rho GTPase activating protein 12	0.624	1.73E-03	2.38E-02
ARHGAP21	Rho GTPase activating protein 21	0.471	1.34E-03	2.07E-02
ARHGEF3	Rho guanine nucleotide exchange factor 3	0.478	5.66E-03	4.63E-02
ARHGEF26	Rho guanine nucleotide exchange factor 26	0.544	2.48E-03	2.89E-02
ARID2	AT-rich interaction domain 2	0.556	5.45E-03	4.53E-02
ARID4A	AT-rich interaction domain 4A	0.656	4.18E-03	3.89E-02
ARL5B	ADP ribosylation factor like GTPase 5B	0.688	3.63E-03	3.59E-02
ARMC1	armadillo repeat containing 1	0.591	3.57E-03	3.57E-02
ARMCX1	armadillo repeat containing, X-linked 1	0.447	5.05E-03	4.34E-02
ASB7	ankyrin repeat and SOCS box containing 7	0.628	2.90E-04	8.79E-03
ASB11	ankyrin repeat and SOCS box containing 11	0.448	3.16E-03	3.33E-02
ASB14	ankyrin repeat and SOCS box containing 14	0.933	1.13E-04	5.23E-03
ASXL3	additional sex combs like 3, transcriptional regulator	0.574	3.01E-04	8.91E-03
ASZ1	ankyrin repeat, SAM and basic leucine zipper domain containing 1	0.975	1.32E-03	2.03E-02
ATAD5	ATPase family, AAA domain containing 5	0.796	8.02E-04	1.54E-02
ATG3	autophagy related 3	0.584	4.31E-03	3.96E-02
ATG14	autophagy related 14	0.545	8.31E-04	1.58E-02
ATL1	atlastin GTPase 1	1.028	2.49E-04	8.23E-03
ATP11B	ATPase phospholipid transporting 11B (putative)	0.592	5.35E-03	4.48E-02
ATP6V0A2	ATPase H+ transporting V0 subunit a2	0.52	5.23E-04	1.19E-02
ATP6V1A	ATPase H+ transporting V1 subunit A	0.577	3.39E-03	3.47E-02
Atp8b5	ATPase, class I, type 8B, member 5	1.162	4.34E-06	1.07E-03
ATPIF1	ATPase inhibitory factor 1	-0.736	4.90E-03	4.27E-02

ATR	ATR serine/threonine kinase	0.711	3.41E-03	3.48E-02
ATXN2	ataxin 2	0.326	6.01E-03	4.81E-02
AURKB	aurora kinase B	-0.702	3.06E-04	9.01E-03
AXDND1	axonemal dynein light chain domain containing 1	1.212	6.13E-05	3.73E-03
B3GALNT2	beta-1,3-N-acetylgalactosaminyltransferase 2	0.47	3.81E-03	3.68E-02
B4GALT6	beta-1,4-galactosyltransferase 6	1.327	3.93E-05	2.87E-03
BAZ1B	bromodomain adjacent to zinc finger domain 1B	0.434	2.92E-04	8.79E-03
BBS9	Bardet-Biedl syndrome 9	0.621	1.81E-03	2.46E-02
BCAS3	BCAS3, microtubule associated cell migration factor	0.403	5.28E-03	4.45E-02
BCAT1	branched chain amino acid transaminase 1	1.025	1.66E-05	2.17E-03
BCAT2	branched chain amino acid transaminase 2	-1.457	2.30E-03	2.76E-02
BGN	biglycan	-1.163	3.87E-03	3.71E-02
BIRC2	baculoviral IAP repeat containing 2	0.789	1.15E-03	1.87E-02
BLM	Bloom syndrome RecQ like helicase	0.737	3.56E-04	9.63E-03
BLVRB	biliverdin reductase B	-1.466	2.06E-03	2.60E-02
BMP1	bone morphogenetic protein 1	-1.675	4.91E-04	1.15E-02
BMPR1B	bone morphogenetic protein receptor type 1B	0.854	3.53E-04	9.58E-03
BMS1	BMS1, ribosome biogenesis factor	0.653	6.68E-04	1.36E-02
BPTF	bromodomain PHD finger transcription factor	0.479	6.49E-04	1.34E-02
BRAF	B-Raf proto-oncogene, serine/threonine kinase	0.604	7.59E-04	1.49E-02
BRAP	BRCA1 associated protein	0.582	1.10E-04	5.15E-03
BRCA1	BRCA1, DNA repair associated	0.671	1.16E-03	1.88E-02
BRCA2	BRCA2, DNA repair associated	0.724	1.54E-03	2.24E-02
BRD7	bromodomain containing 7	0.556	1.54E-03	2.24E-02
BRIX1	BRX1, biogenesis of ribosomes	0.771	8.68E-04	1.60E-02
BTBD8	BTB domain containing 8	1.001	2.63E-04	8.34E-03
BUD13	BUD13 homolog	0.584	1.37E-03	2.08E-02
C3	complement C3	-1.107	1.73E-03	2.38E-02
C10orf76	chromosome 10 open reading frame 76	0.575	1.13E-03	1.85E-02
C11orf57	chromosome 11 open reading frame 57	0.618	9.72E-04	1.70E-02
C11orf70	chromosome 11 open reading frame 70	0.738	3.23E-03	3.38E-02
C11orf71	chromosome 11 open reading frame 71	-1.277	1.66E-03	2.32E-02

C12orf45	chromosome 12 open reading frame 45	0.632	4.54E-03	4.08E-02
C14orf1	chromosome 14 open reading frame 1	-0.917	3.56E-03	3.57E-02
C14orf28	chromosome 14 open reading frame 28	0.589	6.14E-03	4.88E-02
C1orf112	chromosome 1 open reading frame 112	0.819	7.84E-04	1.52E-02
C1orf146	chromosome 1 open reading frame 146	1.153	1.07E-04	5.04E-03
C1QA	complement C1q A chain	-0.955	3.87E-04	9.85E-03
C1RL	complement C1r subcomponent like	-1.662	5.26E-03	4.45E-02
C20orf24	chromosome 20 open reading frame 24	-0.928	1.81E-05	2.23E-03
C20orf194	chromosome 20 open reading frame 194	0.49	4.95E-03	4.30E-02
C4orf33	chromosome 4 open reading frame 33	0.746	3.40E-03	3.48E-02
C4orf47	chromosome 4 open reading frame 47	0.677	2.14E-03	2.66E-02
C6orf62	chromosome 6 open reading frame 62	0.776	1.49E-03	2.20E-02
C7orf31	chromosome 7 open reading frame 31	1.212	2.45E-05	2.36E-03
C9orf84	chromosome 9 open reading frame 84	1.203	3.37E-04	9.43E-03
CA2	carbonic anhydrase 2	-1.082	2.22E-03	2.70E-02
CA10	carbonic anhydrase 10	0.663	3.09E-03	3.30E-02
CAB39	calcium binding protein 39	0.731	1.06E-03	1.79E-02
CACHD1	cache domain containing 1	0.729	1.87E-04	7.00E-03
CASP8AP2	caspase 8 associated protein 2	0.871	3.58E-04	9.63E-03
CBL	Cbl proto-oncogene	0.353	5.59E-03	4.61E-02
CBLB	Cbl proto-oncogene B	0.427	3.78E-03	3.66E-02
CBLL1	Cbl proto-oncogene like 1	0.535	2.53E-03	2.93E-02
CBLN3	cerebellin 3 precursor	-0.936	4.02E-03	3.79E-02
CCAR1	cell division cycle and apoptosis regulator 1	0.768	3.83E-03	3.69E-02
CCBE1	collagen and calcium binding EGF domains 1	-1.284	2.58E-03	2.96E-02
CCDC18	coiled-coil domain containing 18	0.989	2.14E-04	7.41E-03
CCDC30	coiled-coil domain containing 30	0.953	5.45E-05	3.50E-03
CCDC39	coiled-coil domain containing 39	1.338	3.55E-05	2.73E-03
CCDC65	coiled-coil domain containing 65	0.864	4.24E-05	2.99E-03
CCDC66	coiled-coil domain containing 66	0.723	3.89E-03	3.72E-02
CCDC73	coiled-coil domain containing 73	1.52	5.04E-06	1.14E-03
CCDC82	coiled-coil domain containing 82	0.864	7.49E-04	1.48E-02

CCDC112	coiled-coil domain containing 112	0.753	1.66E-03	2.32E-02
CCDC117	coiled-coil domain containing 117	1.109	2.27E-05	2.31E-03
CCDC125	coiled-coil domain containing 125	0.832	1.58E-03	2.27E-02
CCDC150	coiled-coil domain containing 150	0.797	8.82E-05	4.44E-03
CCDC158	coiled-coil domain containing 158	0.779	3.22E-05	2.64E-03
CCDC171	coiled-coil domain containing 171	0.819	6.06E-04	1.30E-02
CCDC181	coiled-coil domain containing 181	1.212	9.98E-06	1.58E-03
CCDC169-SOHLH2	CCDC169-SOHLH2 readthrough	0.86	1.27E-03	1.99E-02
CCNB1IP1	cyclin B1 interacting protein 1	0.808	2.58E-03	2.96E-02
CCNI	cyclin I	0.516	3.34E-03	3.43E-02
CCNL1	cyclin L1	0.597	1.63E-03	2.31E-02
CCNT1	cyclin T1	0.461	4.44E-03	4.02E-02
CCNT2	cyclin T2	0.715	2.57E-03	2.95E-02
CCNYL1	cyclin Y like 1	0.72	5.64E-05	3.59E-03
CCT2	chaperonin containing TCP1 subunit 2	0.594	3.25E-03	3.39E-02
CCT8	chaperonin containing TCP1 subunit 8	0.605	3.98E-03	3.78E-02
CCT6B	chaperonin containing TCP1 subunit 6B	0.772	1.48E-03	2.20E-02
CD34	CD34 molecule	-0.421	4.17E-03	3.88E-02
CD109	CD109 molecule	0.698	1.42E-03	2.14E-02
CDC7	cell division cycle 7	0.654	5.23E-03	4.43E-02
CDC16	cell division cycle 16	0.566	2.49E-03	2.90E-02
CDC23	cell division cycle 23	0.56	5.55E-03	4.60E-02
CDCA7	cell division cycle associated 7	0.522	2.69E-03	3.03E-02
CDCA7L	cell division cycle associated 7 like	0.516	1.76E-04	6.84E-03
CDH2	cadherin 2	0.466	3.35E-03	3.44E-02
CDH11	cadherin 11	-0.452	5.77E-03	4.68E-02
CDK13	cyclin dependent kinase 13	0.459	1.08E-03	1.80E-02
CDK17	cyclin dependent kinase 17	0.683	8.88E-04	1.63E-02
CDKAL1	CDK5 regulatory subunit associated protein 1 like 1	0.636	4.12E-03	3.86E-02
CDKL2	cyclin dependent kinase like 2	0.892	1.75E-03	2.40E-02
Cdkn1c	cyclin-dependent kinase inhibitor 1C (P57)	-1.698	3.16E-03	3.33E-02

CDKN2C	cyclin dependent kinase inhibitor 2C	-0.658	4.05E-04	1.02E-02
CDON	cell adhesion associated, oncogene regulated	0.47	1.48E-04	6.04E-03
CDS1	CDP-diacylglycerol synthase 1	0.93	1.37E-04	5.75E-03
CEMIP	cell migration inducing hyaluronan binding protein	-1.714	1.30E-04	5.60E-03
CENPC	centromere protein C	0.869	4.40E-04	1.08E-02
CENPE	centromere protein E	0.85	1.30E-03	2.01E-02
CENPJ	centromere protein J	0.657	1.36E-03	2.08E-02
CEP63	centrosomal protein 63	0.757	7.90E-04	1.53E-02
CEP83	centrosomal protein 83	0.974	6.77E-05	3.86E-03
CEP85	centrosomal protein 85	0.534	1.07E-03	1.79E-02
CEP112	centrosomal protein 112	0.47	4.06E-03	3.81E-02
CEP120	centrosomal protein 120	0.808	2.33E-03	2.77E-02
CEP135	centrosomal protein 135	1.248	9.51E-05	4.61E-03
CEP290	centrosomal protein 290	0.856	2.00E-03	2.55E-02
CEP295	centrosomal protein 295	0.669	1.94E-03	2.52E-02
CEP350	centrosomal protein 350	0.584	4.16E-03	3.88E-02
CEP85L	centrosomal protein 85 like	0.806	2.81E-04	8.61E-03
CFH	complement factor H	0.755	3.72E-03	3.64E-02
CHAMP1	chromosome alignment maintaining phosphoprotein 1	0.477	1.58E-03	2.27E-02
CHD1	chromodomain helicase DNA binding protein 1	0.624	5.92E-03	4.76E-02
CHI3L1	chitinase 3 like 1	-0.763	1.47E-03	2.19E-02
CHML	CHM like, Rab escort protein 2	0.871	1.79E-04	6.90E-03
CHN1	chimerin 1	0.885	3.41E-07	5.62E-04
CHST15	carbohydrate sulfotransferase 15	-0.974	3.63E-04	9.63E-03
CHSY1	chondroitin sulfate synthase 1	0.58	2.91E-03	3.19E-02
CLASP2	cytoplasmic linker associated protein 2	0.525	2.72E-03	3.05E-02
CLEC14A	C-type lectin domain family 14 member A	-0.98	1.94E-05	2.25E-03
CLIC5	chloride intracellular channel 5	-1.21	3.28E-04	9.32E-03
CLIP1	CAP-Gly domain containing linker protein 1	0.481	2.05E-03	2.58E-02
CLOCK	clock circadian regulator	0.489	3.14E-03	3.33E-02
CLUAP1	clusterin associated protein 1	0.576	1.44E-03	2.17E-02
CMKLR1	chemerin chemokine-like receptor 1	-1.447	2.12E-04	7.41E-03

CMYA5	cardiomyopathy associated 5	0.99	3.53E-05	2.73E-03
CNEP1R1	CTD nuclear envelope phosphatase 1 regulatory subunit 1	0.687	5.06E-03	4.35E-02
CNOT10	CCR4-NOT transcription complex subunit 10	0.555	4.44E-04	1.08E-02
CNP	2',3'-cyclic nucleotide 3' phosphodiesterase	-1.395	6.23E-04	1.32E-02
CNTNAP4	contactin associated protein like 4	1.009	2.00E-03	2.55E-02
COA7	cytochrome c oxidase assembly factor 7 (putative)	0.686	1.96E-04	7.18E-03
COIL	coilin	0.616	5.34E-04	1.20E-02
COL11A1	collagen type XI alpha 1 chain	1.16	4.33E-04	1.07E-02
COL12A1	collagen type XII alpha 1 chain	0.658	5.01E-04	1.17E-02
COL15A1	collagen type XV alpha 1 chain	-0.775	8.70E-06	1.46E-03
COMMD8	COMM domain containing 8	0.967	4.81E-04	1.14E-02
COPG2	coatomer protein complex subunit gamma 2	0.58	2.66E-03	3.02E-02
COX14	COX14, cytochrome c oxidase assembly factor	-0.777	2.32E-03	2.76E-02
COX20	COX20, cytochrome c oxidase assembly factor	0.732	5.59E-03	4.61E-02
COX7A1	cytochrome c oxidase subunit 7A1	-0.773	2.50E-03	2.90E-02
CPA5	carboxypeptidase A5	0.727	1.83E-03	2.47E-02
CPE	carboxypeptidase E	1.049	2.18E-03	2.68E-02
CPN1	carboxypeptidase N subunit 1	0.92	1.14E-04	5.24E-03
CPT2	carnitine palmitoyltransferase 2	-0.523	1.28E-03	2.00E-02
CPT1A	carnitine palmitoyltransferase 1A	-0.548	4.52E-03	4.06E-02
CPXM1	carboxypeptidase X, M14 family member 1	-1.272	2.54E-05	2.36E-03
CRAT	carnitine O-acetyltransferase	-1.315	1.95E-03	2.52E-02
CREBRF	CREB3 regulatory factor	0.755	2.60E-04	8.33E-03
CREM	cAMP responsive element modulator	0.94	3.39E-04	9.46E-03
CRISPLD1	cysteine rich secretory protein LCCL domain containing 1	1.363	9.12E-05	4.52E-03
CRLF3	cytokine receptor like factor 3	0.539	2.87E-03	3.16E-02
CRLS1	cardiolipin synthase 1	0.75	2.89E-03	3.17E-02
CRY1	cryptochrome circadian clock 1	0.718	1.34E-03	2.06E-02
CSDE1	cold shock domain containing E1	0.549	3.89E-03	3.72E-02
CSF1R	colony stimulating factor 1 receptor	-0.846	4.61E-03	4.11E-02
CSGALNACT1	chondroitin sulfate N-acetylgalactosaminyltransferase 1	0.564	1.26E-03	1.98E-02
CSGALNACT2	chondroitin sulfate N-acetylgalactosaminyltransferase 2	0.633	3.15E-03	3.33E-02

CSNK1G3	casein kinase 1 gamma 3	0.774	1.49E-03	2.20E-02
CST9L	cystatin 9-like	-0.831	1.44E-04	5.95E-03
CTCFL	CCCTC-binding factor like	0.937	2.09E-04	7.37E-03
CTNNAL1	catenin alpha like 1	0.787	3.62E-04	9.63E-03
CTPS1	CTP synthase 1	0.489	2.04E-03	2.58E-02
CTSC	cathepsin C	-0.926	2.60E-05	2.39E-03
CTSF	cathepsin F	-1.403	2.33E-05	2.34E-03
CTTNBP2	cortactin binding protein 2	0.493	2.78E-03	3.09E-02
CUL2	cullin 2	0.557	5.69E-03	4.64E-02
CUL3	cullin 3	0.541	5.02E-03	4.33E-02
CWF19L1	CWF19-like 1, cell cycle control (S. pombe)	0.587	3.98E-03	3.78E-02
CXCL9	C-X-C motif chemokine ligand 9	0.91	4.76E-03	4.19E-02
CXorf23	chromosome X open reading frame 23	0.713	4.80E-03	4.20E-02
CXorf38	chromosome X open reading frame 38	0.475	5.67E-03	4.63E-02
CXorf56	chromosome X open reading frame 56	0.532	1.08E-03	1.80E-02
CXorf57	chromosome X open reading frame 57	0.877	3.44E-03	3.49E-02
CYB561	cytochrome b561	-1.081	7.06E-04	1.42E-02
Cyb5r3	cytochrome b5 reductase 3	-1.383	2.82E-03	3.12E-02
CYP11A1	cytochrome P450 family 11 subfamily A member 1	-1.772	6.08E-03	4.85E-02
CYP17A1	cytochrome P450 family 17 subfamily A member 1	-1.378	3.70E-03	3.62E-02
CYP1B1	cytochrome P450 family 1 subfamily B member 1	-0.979	1.40E-03	2.12E-02
CYP27A1	cytochrome P450 family 27 subfamily A member 1	-0.959	1.66E-03	2.32E-02
Cyp2d22	cytochrome P450, family 2, subfamily d, polypeptide 22	-1.925	4.87E-03	4.25E-02
CYP2R1	cytochrome P450 family 2 subfamily R member 1	0.791	1.29E-03	2.01E-02
DAAM2	dishevelled associated activator of morphogenesis 2	-0.991	1.22E-03	1.94E-02
DAZL	deleted in azoospermia like	1.002	8.90E-05	4.46E-03
DBF4	DBF4 zinc finger	0.777	2.67E-03	3.03E-02
DBP	D-box binding PAR bZIP transcription factor	-1.111	7.36E-04	1.46E-02
DCAF12	DDB1 and CUL4 associated factor 12	0.658	2.50E-05	2.36E-03
DCLK2	doublecortin like kinase 2	0.536	3.52E-03	3.55E-02
DCUN1D5	defective in cullin neddylation 1 domain containing 5	0.602	5.09E-03	4.36E-02
DDHD1	DDHD domain containing 1	0.72	1.43E-04	5.93E-03

DDX4	DEAD-box helicase 4	1.304	2.22E-06	8.01E-04
DDX6	DEAD-box helicase 6	0.583	5.12E-03	4.38E-02
DDX10	DEAD-box helicase 10	0.683	3.52E-04	9.58E-03
DDX25	DEAD-box helicase 25	0.781	2.10E-04	7.37E-03
DDX43	DEAD-box helicase 43	1.348	3.56E-06	9.60E-04
DDX50	DExD-box helicase 50	0.54	2.34E-03	2.78E-02
DDX52	DExD-box helicase 52	0.639	3.30E-03	3.41E-02
DDX19B	DEAD-box helicase 19B	-0.47	5.55E-03	4.59E-02
DENND2C	DENN domain containing 2C	1.108	7.83E-05	4.24E-03
DEPDC7	DEP domain containing 7	0.839	3.24E-04	9.24E-03
DEPTOR	DEP domain containing MTOR interacting protein	-1.288	3.07E-05	2.60E-03
DHCR7	7-dehydrocholesterol reductase	-2.552	2.10E-06	7.97E-04
DHCR24	24-dehydrocholesterol reductase	-2.271	5.99E-07	6.25E-04
DHRS4	dehydrogenase/reductase 4	-0.736	3.62E-03	3.58E-02
DHRS12	dehydrogenase/reductase 12	-0.758	1.99E-04	7.18E-03
DHX9	DExH-box helicase 9	0.495	2.93E-03	3.19E-02
DHX32	DEAH-box helicase 32 (putative)	-0.53	2.92E-03	3.19E-02
DHX35	DEAH-box helicase 35	0.583	9.44E-04	1.69E-02
DIP2B	disco interacting protein 2 homolog B	0.425	3.92E-03	3.74E-02
DIS3	DIS3 homolog, exosome endoribonuclease and 3'-5' exoribonuclease	0.784	1.97E-03	2.55E-02
DIS3L	DIS3 like exosome 3'-5' exoribonuclease	0.431	4.82E-03	4.22E-02
DKK2	dickkopf WNT signaling pathway inhibitor 2	-1.653	3.31E-09	3.88E-05
DMTF1	cyclin D binding myb like transcription factor 1	0.785	1.45E-04	5.95E-03
DNAH5	dynein axonemal heavy chain 5	1.139	8.44E-05	4.37E-03
DNAH7	dynein axonemal heavy chain 7	1.237	2.46E-06	8.48E-04
DNAH8	dynein axonemal heavy chain 8	1.418	2.30E-04	7.84E-03
DNAH10	dynein axonemal heavy chain 10	0.941	3.61E-05	2.75E-03
DNAH12	dynein axonemal heavy chain 12	1.019	6.72E-05	3.86E-03
DNAJC2	DnaJ heat shock protein family (Hsp40) member C2	0.783	3.09E-04	9.07E-03
DNAJC6	DnaJ heat shock protein family (Hsp40) member C6	1.244	1.76E-05	2.22E-03
DNAJC21	DnaJ heat shock protein family (Hsp40) member C21	0.669	1.07E-03	1.80E-02
DOCK11	dedicator of cytokinesis 11	0.824	2.51E-04	8.25E-03

DONSON	downstream neighbor of SON	0.584	4.77E-03	4.19E-02
DOPEY1	dopey family member 1	0.628	3.24E-04	9.24E-03
DOPEY2	dopey family member 2	0.446	4.00E-03	3.79E-02
DRC1	dynein regulatory complex subunit 1	0.874	3.53E-04	9.58E-03
DROSHA	drosha ribonuclease III	0.523	4.41E-03	4.02E-02
DSC2	desmocollin 2	0.858	2.81E-04	8.61E-03
DSG2	desmoglein 2	1.011	1.40E-03	2.11E-02
DSN1	DSN1 homolog, MIS12 kinetochore complex component	0.496	3.72E-03	3.64E-02
DUSP1	dual specificity phosphatase 1	-0.57	6.18E-03	4.89E-02
DYRK1A	dual specificity tyrosine phosphorylation regulated kinase 1A	0.488	8.92E-05	4.46E-03
DYX1C1	dyslexia susceptibility 1 candidate 1	0.648	5.95E-03	4.78E-02
DZIP3	DAZ interacting zinc finger protein 3	0.97	1.36E-04	5.71E-03
E2F6	E2F transcription factor 6	0.78	6.65E-06	1.26E-03
EBAG9	estrogen receptor binding site associated, antigen, 9	0.812	3.07E-03	3.29E-02
EBP	emopamil binding protein (sterol isomerase)	-0.95	3.32E-03	3.42E-02
ECD	ecdysoneless cell cycle regulator	0.509	3.74E-03	3.65E-02
ECE1	endothelin converting enzyme 1	-1.152	1.20E-04	5.39E-03
ECH1	enoyl-CoA hydratase 1	-0.544	3.84E-03	3.69E-02
ECHS1	enoyl-CoA hydratase, short chain 1	-0.75	1.99E-04	7.18E-03
EDRF1	erythroid differentiation regulatory factor 1	0.454	4.25E-03	3.94E-02
EEA1	early endosome antigen 1	0.757	3.59E-03	3.58E-02
EEF1B2	eukaryotic translation elongation factor 1 beta 2	0.626	2.19E-03	2.69E-02
EFCAB6	EF-hand calcium binding domain 6	0.636	1.83E-04	6.90E-03
EFEMP1	EGF containing fibulin like extracellular matrix protein 1	-1.268	3.94E-04	9.98E-03
EGFLAM	EGF like, fibronectin type III and laminin G domains	-0.634	2.86E-03	3.15E-02
EIF2B3	eukaryotic translation initiation factor 2B subunit gamma	0.569	5.86E-03	4.72E-02
EIF3M	eukaryotic translation initiation factor 3 subunit M	0.626	3.78E-03	3.66E-02
EIF4EBP2	eukaryotic translation initiation factor 4E binding protein 2	0.524	2.50E-03	2.90E-02
EIF5B	eukaryotic translation initiation factor 5B	0.511	5.14E-03	4.39E-02
ELAVL2	ELAV like RNA binding protein 2	1.101	1.91E-05	2.25E-03
ELK1	ELK1, ETS transcription factor	-0.555	5.04E-03	4.34E-02
ELMO1	engulfment and cell motility 1	0.491	3.17E-03	3.34E-02

ELOVL4	ELOVL fatty acid elongase 4	1.018	6.78E-05	3.86E-03
EMILIN2	elastin microfibril interfacer 2	-1.19	2.63E-04	8.34E-03
EML5	echinoderm microtubule associated protein like 5	0.54	1.69E-03	2.34E-02
EMP2	epithelial membrane protein 2	0.503	4.99E-03	4.32E-02
EMSY	EMSY, BRCA2 interacting transcriptional repressor	0.437	5.60E-03	4.61E-02
ENO1	enolase 1	-0.93	1.81E-03	2.46E-02
ENO4	enolase family member 4	0.588	2.72E-03	3.05E-02
ENOSF1	enolase superfamily member 1	-0.683	1.40E-03	2.11E-02
ENPP2	ectonucleotide pyrophosphatase/phosphodiesterase 2	0.937	3.51E-05	2.73E-03
ENPP3	ectonucleotide pyrophosphatase/phosphodiesterase 3	-1.814	5.84E-04	1.26E-02
ENPP6	ectonucleotide pyrophosphatase/phosphodiesterase 6	-1.066	6.89E-05	3.87E-03
EOGT	EGF domain specific O-linked N-acetylglucosamine transferase	0.928	1.20E-04	5.39E-03
EPB41L3	erythrocyte membrane protein band 4.1 like 3	0.351	5.64E-03	4.63E-02
EPB41L4A	erythrocyte membrane protein band 4.1 like 4A	0.882	7.67E-05	4.21E-03
EPB41L4B	erythrocyte membrane protein band 4.1 like 4B	0.631	1.13E-03	1.85E-02
EPHA5	EPH receptor A5	0.853	3.42E-04	9.51E-03
ERBB4	erb-b2 receptor tyrosine kinase 4	0.526	6.27E-03	4.94E-02
ERCC6L	ERCC excision repair 6 like, spindle assembly checkpoint helicase	0.755	1.57E-03	2.27E-02
ERGIC1	endoplasmic reticulum-golgi intermediate compartment 1	-0.782	3.25E-03	3.39E-02
ERO1A	endoplasmic reticulum oxidoreductase 1 alpha	0.769	2.20E-03	2.69E-02
ERRFI1	ERBB receptor feedback inhibitor 1	-0.539	1.04E-03	1.77E-02
ESF1	ESF1 nucleolar pre-rRNA processing protein homolog	0.957	1.78E-04	6.88E-03
ESRP1	epithelial splicing regulatory protein 1	0.904	2.99E-04	8.88E-03
ETAA1	Ewing tumor associated antigen 1	0.64	4.10E-03	3.85E-02
ETV5	ETS variant 5	0.551	9.62E-04	1.70E-02
EXD1	exonuclease 3'-5' domain containing 1	0.975	2.41E-04	8.04E-03
EXOSC8	exosome component 8	0.802	1.26E-03	1.98E-02
EYA1	EYA transcriptional coactivator and phosphatase 1	1.026	9.48E-05	4.61E-03
EYA4	EYA transcriptional coactivator and phosphatase 4	0.965	4.43E-05	3.04E-03
F13A1	coagulation factor XIII A chain	-1.166	1.72E-03	2.37E-02
FADS1	fatty acid desaturase 1	-0.848	6.31E-03	4.95E-02
FADS2	fatty acid desaturase 2	-1.09	2.27E-03	2.73E-02

FAM107B	family with sequence similarity 107 member B	0.721	3.22E-04	9.24E-03
FAM126B	family with sequence similarity 126 member B	0.609	4.42E-03	4.02E-02
FAM129A	family with sequence similarity 129 member A	-0.926	1.78E-06	7.65E-04
FAM129B	family with sequence similarity 129 member B	-1.812	8.51E-04	1.59E-02
FAM166B	family with sequence similarity 166 member B	-1.497	6.29E-06	1.26E-03
FAM169A	family with sequence similarity 169 member A	0.867	2.14E-05	2.31E-03
FAM175A	family with sequence similarity 175 member A	0.584	2.76E-03	3.08E-02
FAM184A	family with sequence similarity 184 member A	1.08	3.04E-06	9.41E-04
FAM186B	family with sequence similarity 186 member B	0.411	2.04E-03	2.58E-02
FAM188A	family with sequence similarity 188 member A	0.664	2.63E-03	3.00E-02
FAM221A	family with sequence similarity 221 member A	1.374	4.03E-06	1.05E-03
FAM227A	family with sequence similarity 227 member A	0.949	2.63E-03	3.00E-02
FAM46D	family with sequence similarity 46 member D	1.053	8.35E-04	1.58E-02
FAM60A	family with sequence similarity 60 member A	0.813	6.38E-04	1.34E-02
FAM76B	family with sequence similarity 76 member B	0.708	2.18E-03	2.68E-02
FAM92A	family with sequence similarity 92 member A	0.689	2.21E-03	2.70E-02
FANCC	Fanconi anemia complementation group C	0.561	4.01E-04	1.01E-02
FANCD2	Fanconi anemia complementation group D2	0.82	2.48E-04	8.20E-03
FANCI	Fanconi anemia complementation group I	0.78	2.05E-04	7.29E-03
FASTKD3	FAST kinase domains 3	0.711	1.03E-03	1.76E-02
FBN2	fibrillin 2	0.571	4.14E-03	3.87E-02
FBXL20	F-box and leucine rich repeat protein 20	0.564	1.13E-03	1.85E-02
FBXL21	F-box and leucine rich repeat protein 21 (gene/pseudogene)	0.813	1.86E-03	2.49E-02
FBXW7	F-box and WD repeat domain containing 7	0.873	1.67E-06	7.65E-04
FCF1	FCF1 rRNA-processing protein	0.6	2.83E-03	3.13E-02
FCGRT	Fc fragment of IgG receptor and transporter	-1.163	3.41E-05	2.70E-03
FCHSD2	FCH and double SH3 domains 2	0.506	2.10E-03	2.62E-02
FDPS	farnesyl diphosphate synthase	-1.169	3.42E-03	3.48E-02
FGFR1OP	FGFR1 oncogene partner	0.421	3.78E-03	3.66E-02
FHDC1	FH2 domain containing 1	-0.754	2.83E-03	3.13E-02
FHL1	four and a half LIM domains 1	0.878	6.49E-04	1.34E-02
FIBIN	fin bud initiation factor homolog (zebrafish)	-0.993	3.59E-03	3.58E-02

Fip1l1	FIP1 like 1 (S. cerevisiae)	0.536	1.04E-03	1.77E-02
FLRT2	fibronectin leucine rich transmembrane protein 2	-0.731	2.62E-03	2.99E-02
FMNL2	formin like 2	0.558	5.12E-03	4.38E-02
FMR1	fragile X mental retardation 1	1.165	1.60E-06	7.65E-04
FMR1NB	fragile X mental retardation 1 neighbor	0.936	4.48E-05	3.04E-03
Fnbp1l	formin binding protein 1-like	0.552	3.48E-03	3.52E-02
FOLR2	folate receptor beta	-0.884	6.05E-03	4.84E-02
FOS	Fos proto-oncogene, AP-1 transcription factor subunit	-1.124	1.62E-04	6.44E-03
FOSL2	FOS like 2, AP-1 transcription factor subunit	-1.625	2.26E-05	2.31E-03
Foxp1	forkhead box P1	0.425	2.11E-03	2.63E-02
FOXRED1	FAD dependent oxidoreductase domain containing 1	-0.799	3.55E-03	3.57E-02
FREM2	FRAS1 related extracellular matrix protein 2	0.495	5.07E-03	4.35E-02
FSD1L	fibronectin type III and SPRY domain containing 1 like	0.809	2.88E-04	8.77E-03
FSHR	follicle stimulating hormone receptor	1.021	3.22E-05	2.64E-03
FSIP2	fibrous sheath interacting protein 2	1.759	4.46E-06	1.07E-03
FYTTD1	forty-two-three domain containing 1	0.568	2.35E-03	2.78E-02
G2E3	G2/M-phase specific E3 ubiquitin protein ligase	0.836	1.08E-03	1.80E-02
GAB3	GRB2 associated binding protein 3	-1.149	2.54E-04	8.25E-03
GADD45B	growth arrest and DNA damage inducible beta	-1.32	2.93E-04	8.80E-03
GART	phosphoribosylglycinamide formyltransferase, phosphoribosylglycinamide synthetase, phosphoribosylaminoimidazole synthetase	0.586	1.86E-04	6.97E-03
GATM	glycine amidinotransferase	0.764	9.67E-04	1.70E-02
GBA	glucosylceramidase beta	-0.513	3.50E-03	3.53E-02
GCA	grancalcin	0.927	7.54E-04	1.48E-02
GDAP1	ganglioside induced differentiation associated protein 1	0.669	5.27E-03	4.45E-02
GEMIN5	gem nuclear organelle associated protein 5	0.464	4.42E-03	4.02E-02
GFPT2	glutamine-fructose-6-phosphate transaminase 2	-0.718	4.23E-05	2.99E-03
GIF	gastric intrinsic factor	1.121	6.48E-04	1.34E-02
GIGYF2	GRB10 interacting GYF protein 2	0.3	4.28E-03	3.95E-02
GIN1	gypsy retrotransposon integrase 1	0.86	2.98E-04	8.88E-03
GKAP1	G kinase anchoring protein 1	1.019	1.15E-04	5.25E-03

GLDC	glycine decarboxylase	0.784	1.06E-04	5.04E-03
GLUL	glutamate-ammonia ligase	-0.847	5.63E-06	1.21E-03
GMCL1	germ cell-less, spermatogenesis associated 1	0.808	9.49E-04	1.69E-02
GMPS	guanine monophosphate synthase	0.544	2.68E-03	3.03E-02
GNAL	G protein subunit alpha L	0.712	1.14E-03	1.86E-02
GNG4	G protein subunit gamma 4	0.942	9.54E-04	1.69E-02
GNL2	G protein nucleolar 2	0.543	1.99E-05	2.25E-03
GNL3	G protein nucleolar 3	0.663	3.62E-03	3.58E-02
GOLM1	golgi membrane protein 1	-0.488	5.01E-03	4.33E-02
GPBP1	GC-rich promoter binding protein 1	0.666	6.16E-03	4.88E-02
GPBP1L1	GC-rich promoter binding protein 1 like 1	0.463	8.09E-04	1.55E-02
GPC3	glypican 3	-1.216	4.11E-04	1.02E-02
GPSM2	G-protein signaling modulator 2	0.644	5.08E-03	4.36E-02
GPT2	glutamic--pyruvic transaminase 2	-0.873	8.09E-04	1.55E-02
GRK5	G protein-coupled receptor kinase 5	-0.729	1.44E-03	2.17E-02
GRPEL1	GrpE like 1, mitochondrial	-0.674	5.37E-03	4.49E-02
GSS	glutathione synthetase	-0.716	1.83E-03	2.47E-02
GSTM4	glutathione S-transferase mu 4	0.837	1.93E-03	2.51E-02
GTF2A1	general transcription factor IIA subunit 1	0.416	2.80E-03	3.10E-02
GTF2H1	general transcription factor IIH subunit 1	0.736	2.42E-04	8.04E-03
GTF3C3	general transcription factor IIIC subunit 3	0.71	1.32E-03	2.04E-02
GTPBP4	GTP binding protein 4	0.438	1.21E-03	1.93E-02
GTSF1	gametocyte specific factor 1	1.123	8.36E-06	1.42E-03
GUCY1B3	guanylate cyclase 1 soluble subunit beta	0.697	2.54E-03	2.93E-02
GUCY2C	guanylate cyclase 2C	1.141	1.82E-06	7.65E-04
HAUS2	HAUS augmin like complex subunit 2	0.811	5.08E-04	1.17E-02
HAUS6	HAUS augmin like complex subunit 6	0.715	3.00E-03	3.25E-02
HBEGF	heparin binding EGF like growth factor	0.856	2.51E-03	2.92E-02
HCK	HCK proto-oncogene, Src family tyrosine kinase	-0.733	5.68E-03	4.63E-02
HDDC3	HD domain containing 3	-0.656	1.80E-03	2.45E-02
HDGFRP3	hepatoma-derived growth factor, related protein 3	0.699	4.56E-04	1.10E-02
HECTD1	HECT domain E3 ubiquitin protein ligase 1	0.5	5.28E-03	4.45E-02

HECTD2	HECT domain E3 ubiquitin protein ligase 2	1.067	1.29E-04	5.59E-03
HELLS	helicase, lymphoid-specific	0.952	1.88E-04	7.00E-03
HENMT1	HEN1 methyltransferase homolog 1	0.684	1.15E-03	1.87E-02
HERC5	HECT and RLD domain containing E3 ubiquitin protein ligase 5	0.892	2.75E-04	8.52E-03
HEXIM1	hexamethylene bisacetamide inducible 1	-0.725	2.44E-03	2.86E-02
HEY2	hes related family bHLH transcription factor with YRPW motif 2	0.761	2.03E-03	2.58E-02
HFM1	HFM1, ATP dependent DNA helicase homolog	1.693	3.37E-06	9.60E-04
HJURP	Holliday junction recognition protein	-0.957	6.33E-04	1.34E-02
HMCN1	hemicentin 1	0.738	1.88E-03	2.49E-02
HMGB3	high mobility group box 3	0.945	1.71E-06	7.65E-04
HMOX1	heme oxygenase 1	-0.948	1.51E-03	2.21E-02
HNRNPA1	heterogeneous nuclear ribonucleoprotein A1	0.447	3.56E-03	3.57E-02
HNRNPDL	heterogeneous nuclear ribonucleoprotein D like	0.611	1.83E-03	2.47E-02
HNRNPH1	heterogeneous nuclear ribonucleoprotein H1	0.568	4.60E-03	4.11E-02
HOOK1	hook microtubule tethering protein 1	0.694	3.36E-04	9.43E-03
HORMAD1	HORMA domain containing 1	1.121	1.19E-04	5.37E-03
HP1BP3	heterochromatin protein 1 binding protein 3	0.552	3.47E-04	9.57E-03
HSD17B10	hydroxysteroid 17-beta dehydrogenase 10	-0.898	1.64E-03	2.31E-02
HSD3B2	hydroxy-delta-5-steroid dehydrogenase, 3 beta- and steroid delta-isomerase 2	-1.332	4.81E-04	1.14E-02
HSD3B7	hydroxy-delta-5-steroid dehydrogenase, 3 beta- and steroid delta-isomerase 7	-1.238	5.88E-04	1.27E-02
HSF2	heat shock transcription factor 2	0.905	4.23E-05	2.99E-03
HSPA12A	heat shock protein family A (Hsp70) member 12A	-0.699	1.88E-03	2.49E-02
HSPA4L	heat shock protein family A (Hsp70) member 4 like	1.012	5.44E-04	1.22E-02
HSPB6	heat shock protein family B (small) member 6	-0.756	2.68E-04	8.42E-03
HSPH1	heat shock protein family H (Hsp110) member 1	0.752	1.27E-03	1.99E-02
HTATSF1	HIV-1 Tat specific factor 1	1.018	2.46E-05	2.36E-03
HTRA1	HtrA serine peptidase 1	-0.693	5.90E-05	3.66E-03
HYDIN	HYDIN, axonemal central pair apparatus protein	0.834	3.18E-04	9.19E-03
HYLS1	HYLS1, centriolar and ciliogenesis associated	0.546	3.45E-03	3.50E-02
IAH1	isoamyl acetate-hydrolyzing esterase 1 homolog	-0.501	5.02E-03	4.33E-02
ICA1L	islet cell autoantigen 1 like	0.913	9.53E-04	1.69E-02
ICAM1	intercellular adhesion molecule 1	-0.766	1.05E-03	1.78E-02

IDNK	IDNK, gluconokinase	0.935	2.55E-04	8.25E-03
IFI30	IFI30, lysosomal thiol reductase	-0.796	1.22E-03	1.93E-02
IFT74	intraflagellar transport 74	0.677	5.58E-03	4.61E-02
IFT81	intraflagellar transport 81	0.823	8.47E-04	1.59E-02
IFT88	intraflagellar transport 88	0.555	1.59E-03	2.28E-02
IGF2BP2	insulin like growth factor 2 mRNA binding protein 2	0.842	2.24E-05	2.31E-03
IGF2BP3	insulin like growth factor 2 mRNA binding protein 3	0.971	1.64E-04	6.47E-03
IGFBP4	insulin like growth factor binding protein 4	-1.049	1.57E-06	7.65E-04
IK	IK cytokine, down-regulator of HLA II	0.474	1.21E-03	1.93E-02
IMPACT	impact RWD domain protein	0.729	1.56E-03	2.26E-02
IMPG2	interphotoreceptor matrix proteoglycan 2	0.624	3.64E-03	3.59E-02
INA	internexin neuronal intermediate filament protein alpha	1	5.65E-04	1.24E-02
INO80	INO80 complex subunit	0.339	4.26E-03	3.94E-02
INSIG1	insulin induced gene 1	-0.958	2.38E-03	2.81E-02
INSL3	insulin like 3	-1.78	1.10E-04	5.15E-03
INTS2	integrator complex subunit 2	0.637	1.75E-03	2.40E-02
INTS6	integrator complex subunit 6	0.713	6.14E-04	1.31E-02
INTS7	integrator complex subunit 7	0.456	4.59E-03	4.11E-02
IPCEF1	interaction protein for cytohesin exchange factors 1	0.832	3.33E-05	2.66E-03
IQCA1	IQ motif containing with AAA domain 1	0.634	2.21E-03	2.69E-02
IQCB1	IQ motif containing B1	1.047	1.55E-05	2.04E-03
IQCG	IQ motif containing G	0.943	8.05E-05	4.28E-03
IQCK	IQ motif containing K	0.475	2.64E-03	3.01E-02
IRAK1BP1	interleukin 1 receptor associated kinase 1 binding protein 1	0.616	2.98E-04	8.88E-03
ISCU	iron-sulfur cluster assembly enzyme	-0.745	4.89E-06	1.13E-03
ITGA2	integrin subunit alpha 2	0.926	1.82E-04	6.90E-03
ITGA6	integrin subunit alpha 6	0.665	4.11E-03	3.85E-02
ITGA9	integrin subunit alpha 9	-0.778	2.26E-05	2.31E-03
ITIH2	inter-alpha-trypsin inhibitor heavy chain 2	0.985	3.14E-03	3.33E-02
ITPR1	inositol 1,4,5-trisphosphate receptor type 1	-0.622	1.40E-04	5.84E-03
IVD	isovaleryl-CoA dehydrogenase	-0.508	4.35E-03	3.99E-02
IVNS1ABP	influenza virus NS1A binding protein	0.634	4.68E-03	4.15E-02

JAK2	Janus kinase 2	0.784	1.12E-03	1.84E-02
JAK3	Janus kinase 3	-1.131	6.43E-04	1.34E-02
JPH1	junctophilin 1	0.714	4.58E-05	3.09E-03
KANSL1	KAT8 regulatory NSL complex subunit 1	0.371	4.42E-03	4.02E-02
KANSL2	KAT8 regulatory NSL complex subunit 2	0.469	1.98E-03	2.55E-02
KANSL1L	KAT8 regulatory NSL complex subunit 1 like	0.709	3.20E-03	3.35E-02
KAT7	lysine acetyltransferase 7	0.499	2.71E-03	3.05E-02
KCND3	potassium voltage-gated channel subfamily D member 3	-1.181	6.16E-03	4.88E-02
KCNE4	potassium voltage-gated channel subfamily E regulatory subunit 4	-1.752	4.82E-05	3.22E-03
KCTD6	potassium channel tetramerization domain containing 6	0.552	3.65E-03	3.59E-02
KCTD20	potassium channel tetramerization domain containing 20	0.46	2.89E-03	3.17E-02
KDM1A	lysine demethylase 1A	0.501	3.80E-04	9.79E-03
KDM3A	lysine demethylase 3A	0.615	3.70E-04	9.73E-03
KDM3B	lysine demethylase 3B	0.358	5.29E-03	4.46E-02
KDM6A	lysine demethylase 6A	0.522	5.04E-03	4.34E-02
KHDRBS3	KH RNA binding domain containing, signal transduction associated 3	0.831	2.53E-04	8.25E-03
KIAA0753	KIAA0753	0.593	1.89E-05	2.25E-03
KIAA1324	KIAA1324	-0.732	6.22E-03	4.91E-02
KIF15	kinesin family member 15	0.78	1.99E-04	7.18E-03
KIF27	kinesin family member 27	0.857	2.64E-04	8.36E-03
KIF18A	kinesin family member 18A	0.89	1.03E-03	1.76E-02
KIZ	kizuna centrosomal protein	0.623	1.99E-04	7.18E-03
KLF9	Kruppel like factor 9	-1.434	1.28E-03	1.99E-02
KLHDC1	kelch domain containing 1	0.917	2.04E-03	2.58E-02
KLHL8	kelch like family member 8	0.732	1.03E-03	1.76E-02
KLHL13	kelch like family member 13	0.657	1.87E-03	2.49E-02
KMT2A	lysine methyltransferase 2A	0.479	2.69E-04	8.42E-03
KMT2E	lysine methyltransferase 2E	0.723	9.00E-05	4.48E-03
KMT5B	lysine methyltransferase 5B	0.601	3.98E-03	3.78E-02
KNTC1	kinetochore associated 1	0.828	2.10E-04	7.37E-03
KPNA3	karyopherin subunit alpha 3	0.611	5.23E-03	4.43E-02
KPNA5	karyopherin subunit alpha 5	0.778	3.61E-03	3.58E-02

KRTCAP2	keratinocyte associated protein 2	-0.493	2.00E-03	2.55E-02
Ktn1	kinectin 1	0.728	1.93E-03	2.51E-02
LAMA3	laminin subunit alpha 3	0.723	3.88E-04	9.86E-03
LARP7	La ribonucleoprotein domain family member 7	0.986	8.70E-05	4.43E-03
LARP1B	La ribonucleoprotein domain family member 1B	0.703	3.04E-03	3.28E-02
LCA5	LCA5, lebercilin	0.825	1.08E-03	1.80E-02
LDLR	low density lipoprotein receptor	-1.789	2.39E-04	8.03E-03
LEF1	lymphoid enhancer binding factor 1	0.867	4.90E-05	3.25E-03
LGALS8	galectin 8	0.351	4.32E-03	3.97E-02
LGALS3BP	galectin 3 binding protein	-0.977	4.46E-03	4.03E-02
LIMCH1	LIM and calponin homology domains 1	0.723	1.59E-03	2.27E-02
LIN52	lin-52 DREAM MuvB core complex component	0.642	9.70E-04	1.70E-02
LINS1	lines homolog 1	0.782	2.42E-03	2.85E-02
LMO7	LIM domain 7	0.713	9.52E-04	1.69E-02
LNX1	ligand of numb-protein X 1	0.736	5.07E-03	4.35E-02
LONRF1	LON peptidase N-terminal domain and ring finger 1	0.73	7.90E-04	1.53E-02
LPAR1	lysophosphatidic acid receptor 1	-0.821	1.13E-04	5.23E-03
LPIN1	lipin 1	-1.047	1.82E-03	2.47E-02
LRP2BP	LRP2 binding protein	0.616	1.63E-03	2.31E-02
LRR1	leucine rich repeat protein 1	0.654	5.10E-03	4.37E-02
LRRC6	leucine rich repeat containing 6	1.09	9.65E-07	7.08E-04
LRRC17	leucine rich repeat containing 17	0.869	1.89E-03	2.50E-02
LRRC42	leucine rich repeat containing 42	0.468	2.79E-03	3.10E-02
LRRC49	leucine rich repeat containing 49	0.845	8.34E-04	1.58E-02
LRRCC1	leucine rich repeat and coiled-coil centrosomal protein 1	0.797	3.34E-03	3.43E-02
LSG1	large 60S subunit nuclear export GTPase 1	0.503	5.13E-04	1.18E-02
LSS	lanosterol synthase	-2.087	3.44E-06	9.60E-04
LY6G5B	lymphocyte antigen 6 family member G5B	0.443	2.89E-03	3.17E-02
LYST	lysosomal trafficking regulator	0.695	2.23E-03	2.70E-02
LZTFL1	leucine zipper transcription factor like 1	0.604	1.31E-03	2.03E-02
MAEL	maelstrom spermatogenic transposon silencer	1.035	7.20E-05	4.01E-03
MAGEB2	MAGE family member B2	0.759	1.70E-03	2.36E-02

MAGEB17	MAGE family member B17	0.645	5.33E-03	4.48E-02
MAGI3	membrane associated guanylate kinase, WW and PDZ domain containing 3	0.625	4.30E-04	1.06E-02
MAK16	MAK16 homolog	0.562	4.89E-03	4.26E-02
MAL2	mal, T-cell differentiation protein 2 (gene/pseudogene)	0.952	3.10E-03	3.30E-02
MALT1	MALT1 paracaspase	0.541	5.35E-03	4.48E-02
MAMSTR	MEF2 activating motif and SAP domain containing transcriptional regulator	-0.689	2.00E-03	2.55E-02
MAN2B1	mannosidase alpha class 2B member 1	-0.835	6.55E-04	1.35E-02
MAP3K1	mitogen-activated protein kinase kinase kinase 1	0.519	2.71E-03	3.05E-02
MAP3K7	mitogen-activated protein kinase kinase kinase 7	0.538	5.65E-03	4.63E-02
MAP3K8	mitogen-activated protein kinase kinase kinase 8	0.688	1.04E-03	1.77E-02
MAP4K3	mitogen-activated protein kinase kinase kinase kinase 3	0.905	1.92E-04	7.10E-03
MAP7D3	MAP7 domain containing 3	0.488	4.31E-03	3.96E-02
MAPK8	mitogen-activated protein kinase 8	0.655	5.76E-03	4.68E-02
MASP2	mannan binding lectin serine peptidase 2	0.928	5.47E-04	1.22E-02
MATN2	matrilin 2	-0.628	2.06E-04	7.32E-03
MBIP	MAP3K12 binding inhibitory protein 1	0.702	2.05E-03	2.59E-02
MBLAC2	metallo-beta-lactamase domain containing 2	0.834	3.29E-03	3.41E-02
MBTD1	mbt domain containing 1	0.733	6.17E-04	1.32E-02
MCM6	minichromosome maintenance complex component 6	0.598	1.64E-03	2.31E-02
MCM8	minichromosome maintenance 8 homologous recombination repair factor	0.8	4.91E-04	1.15E-02
MCM9	minichromosome maintenance 9 homologous recombination repair factor	0.49	1.01E-03	1.74E-02
MCM10	minichromosome maintenance 10 replication initiation factor	0.803	2.77E-04	8.55E-03
MCTS1	MCTS1, re-initiation and release factor	0.736	6.25E-03	4.93E-02
MCUR1	mitochondrial calcium uniporter regulator 1	0.602	4.31E-03	3.96E-02
MDM1	Mdm1 nuclear protein	0.663	4.47E-03	4.04E-02
MDN1	midasin AAA ATPase 1	0.497	1.58E-04	6.33E-03
MEAF6	MYST/Esa1 associated factor 6	0.492	3.31E-03	3.42E-02
MED14	mediator complex subunit 14	0.738	2.38E-05	2.34E-03
MED18	mediator complex subunit 18	0.451	4.21E-03	3.91E-02
MED12L	mediator complex subunit 12 like	0.533	1.01E-03	1.74E-02
MED13L	mediator complex subunit 13 like	0.405	9.37E-04	1.68E-02
MEF2A	myocyte enhancer factor 2A	0.589	4.25E-03	3.94E-02

METAP1D	methionyl aminopeptidase type 1D, mitochondrial	0.502	4.36E-03	3.99E-02
METTL6	methyltransferase like 6	0.643	1.26E-03	1.98E-02
METTL16	methyltransferase like 16	0.498	3.23E-03	3.38E-02
MFSD1	major facilitator superfamily domain containing 1	0.66	1.17E-03	1.88E-02
MFSD4B	major facilitator superfamily domain containing 4B	0.546	5.50E-03	4.57E-02
MGAT1	mannosyl (alpha-1,3-)-glycoprotein beta-1,2-N-acetylglucosaminyltransferase	-0.493	3.97E-03	3.77E-02
MGEA5	meningioma expressed antigen 5 (hyaluronidase)	0.57	4.64E-03	4.13E-02
MIS18BP1	MIS18 binding protein 1	0.976	2.02E-04	7.22E-03
MKNK1	MAP kinase interacting serine/threonine kinase 1	-0.409	6.27E-03	4.94E-02
MLF1	myeloid leukemia factor 1	0.688	6.57E-04	1.35E-02
MLH1	mutL homolog 1	0.362	1.52E-03	2.22E-02
MLLT10	myeloid/lymphoid or mixed-lineage leukemia; translocated to, 10	0.503	1.78E-04	6.88E-03
MMAB	methylmalonic aciduria (cobalamin deficiency) cblB type	-1.169	3.90E-05	2.86E-03
MND1	meiotic nuclear divisions 1	0.809	3.13E-04	9.12E-03
MNS1	meiosis specific nuclear structural 1	0.851	1.60E-03	2.28E-02
MOB1B	MOB kinase activator 1B	0.478	4.99E-03	4.32E-02
MORC1	MORC family CW-type zinc finger 1	1.425	1.36E-05	1.92E-03
MORC3	MORC family CW-type zinc finger 3	0.584	5.46E-03	4.54E-02
MPDZ	multiple PDZ domain crumbs cell polarity complex component	0.652	2.16E-03	2.67E-02
MPHOSPH6	M-phase phosphoprotein 6	0.887	4.35E-04	1.07E-02
MPHOSPH8	M-phase phosphoprotein 8	0.438	6.37E-03	4.99E-02
MPHOSPH10	M-phase phosphoprotein 10	0.639	2.91E-04	8.79E-03
MPP1	membrane palmitoylated protein 1	-1.328	1.94E-06	7.83E-04
MPP6	membrane palmitoylated protein 6	0.629	5.63E-03	4.62E-02
MPP7	membrane palmitoylated protein 7	1.094	6.60E-05	3.86E-03
MRE11	MRE11 homolog, double strand break repair nuclease	0.669	3.09E-03	3.30E-02
MRPL17	mitochondrial ribosomal protein L17	-0.701	2.83E-03	3.13E-02
MRPL32	mitochondrial ribosomal protein L32	0.574	5.04E-04	1.17E-02
MRPS15	mitochondrial ribosomal protein S15	0.442	3.28E-03	3.41E-02
MRPS25	mitochondrial ribosomal protein S25	-0.852	1.68E-03	2.34E-02
MSL1	male specific lethal 1 homolog	0.609	3.14E-04	9.13E-03
MSL2	male-specific lethal 2 homolog (Drosophila)	0.536	4.46E-03	4.03E-02

MSL3	male-specific lethal 3 homolog (Drosophila)	0.532	5.15E-03	4.39E-02
MSLN	mesothelin	-1.644	1.92E-03	2.51E-02
MTBP	MDM2 binding protein	0.739	1.34E-03	2.06E-02
MTIF2	mitochondrial translational initiation factor 2	0.746	1.91E-03	2.51E-02
MTSS1	MTSS1, I-BAR domain containing	0.448	9.35E-04	1.68E-02
MTTP	microsomal triglyceride transfer protein	1.082	2.61E-04	8.33E-03
MTURN	maturin, neural progenitor differentiation regulator homolog	0.996	3.83E-04	9.79E-03
MVD	mevalonate diphosphate decarboxylase	-2.726	8.71E-05	4.43E-03
MVK	mevalonate kinase	-1.91	2.87E-04	8.75E-03
MXI1	MAX interactor 1, dimerization protein	0.666	4.02E-03	3.79E-02
MYCBP2	MYC binding protein 2, E3 ubiquitin protein ligase	0.534	6.79E-04	1.37E-02
MYH1	myosin heavy chain 1	0.827	3.90E-03	3.72E-02
MYH15	myosin heavy chain 15	0.855	4.29E-04	1.06E-02
MYLIP	myosin regulatory light chain interacting protein	0.542	1.23E-03	1.95E-02
MYO1A	myosin IA	1.115	6.52E-06	1.26E-03
MYO1B	myosin IB	0.691	1.48E-03	2.19E-02
MYO1E	myosin IE	0.728	2.85E-06	9.04E-04
N6AMT1	N-6 adenine-specific DNA methyltransferase 1 (putative)	0.902	2.51E-05	2.36E-03
NAA11	N(alpha)-acetyltransferase 11, NatA catalytic subunit	0.796	5.59E-03	4.61E-02
NAA15	N(alpha)-acetyltransferase 15, NatA auxiliary subunit	0.642	3.85E-03	3.70E-02
NAB1	NGFI-A binding protein 1	0.627	3.00E-03	3.25E-02
NALCN	sodium leak channel, non-selective	0.628	5.35E-03	4.48E-02
NANP	N-acetylneuraminic acid phosphatase	0.62	4.24E-03	3.94E-02
NAP1L1	nucleosome assembly protein 1 like 1	0.702	5.14E-03	4.39E-02
NAPB	NSF attachment protein beta	0.445	3.81E-04	9.79E-03
NASP	nuclear autoantigenic sperm protein	0.552	8.49E-04	1.59E-02
NBAS	neuroblastoma amplified sequence	0.362	1.85E-03	2.48E-02
NBEA	neurobeachin	0.672	9.41E-05	4.61E-03
NCAPG	non-SMC condensin I complex subunit G	0.676	3.29E-03	3.41E-02
NCBP1	nuclear cap binding protein subunit 1	0.583	5.92E-03	4.76E-02
NCEH1	neutral cholesterol ester hydrolase 1	0.754	3.34E-03	3.43E-02
NDRG1	N-myc downstream regulated 1	-0.656	4.86E-04	1.15E-02

NDUFA6	NADH:ubiquinone oxidoreductase subunit A6	-0.449	2.91E-03	3.18E-02
NDUFA10	NADH:ubiquinone oxidoreductase subunit A10	-0.619	6.33E-03	4.96E-02
NDUFAF2	NADH:ubiquinone oxidoreductase complex assembly factor 2	0.774	1.58E-04	6.33E-03
NDUFB1	NADH:ubiquinone oxidoreductase subunit B1	-0.591	4.10E-04	1.02E-02
NEB	nebulin	0.77	2.38E-03	2.81E-02
NECAP2	NECAP endocytosis associated 2	-0.764	3.31E-03	3.42E-02
NECTIN3	nectin cell adhesion molecule 3	0.667	5.70E-03	4.64E-02
NEDD4L	neural precursor cell expressed, developmentally down-regulated 4-like, E3 ubiquitin protein ligase	0.378	4.70E-03	4.16E-02
NEK1	NIMA related kinase 1	0.514	2.07E-03	2.60E-02
NEK3	NIMA related kinase 3	0.617	3.93E-03	3.75E-02
NEK6	NIMA related kinase 6	0.743	8.05E-05	4.28E-03
NEK9	NIMA related kinase 9	0.317	5.60E-03	4.61E-02
NELL2	neural EGFL like 2	0.975	3.58E-04	9.63E-03
NEMF	nuclear export mediator factor	0.662	4.49E-03	4.05E-02
NEPRO	nucleolus and neural progenitor protein	0.721	2.72E-03	3.05E-02
NFATC3	nuclear factor of activated T-cells 3	0.532	5.55E-04	1.23E-02
NFE2L1	nuclear factor, erythroid 2 like 1	-0.639	6.75E-04	1.37E-02
NFIL3	nuclear factor, interleukin 3 regulated	0.603	4.68E-03	4.14E-02
NFXL1	nuclear transcription factor, X-box binding like 1	0.729	6.93E-04	1.40E-02
NGDN	neuroguidin	0.492	5.58E-03	4.61E-02
NIFK	nucleolar protein interacting with the FHA domain of MKI67	0.592	3.53E-03	3.56E-02
NKIRAS2	NFKB inhibitor interacting Ras like 2	-0.995	1.90E-03	2.50E-02
NKRF	NFKB repressing factor	1.062	1.18E-04	5.37E-03
NME2	NME/NM23 nucleoside diphosphate kinase 2	-1.294	8.21E-05	4.34E-03
NMT2	N-myristoyltransferase 2	0.807	9.78E-06	1.57E-03
NOL3	nucleolar protein 3	-1.8	5.77E-03	4.68E-02
NOL11	nucleolar protein 11	0.551	4.13E-03	3.86E-02
NOP58	NOP58 ribonucleoprotein	0.713	7.13E-04	1.42E-02
NOV	nephroblastoma overexpressed	1.683	3.49E-03	3.53E-02
NPC1	NPC intracellular cholesterol transporter 1	-0.845	4.59E-04	1.10E-02

NPNT	nephronectin	0.835	3.80E-04	9.79E-03
NR1D1	nuclear receptor subfamily 1 group D member 1	-1.141	2.97E-03	3.22E-02
NR3C2	nuclear receptor subfamily 3 group C member 2	0.636	1.76E-03	2.41E-02
NR6A1	nuclear receptor subfamily 6 group A member 1	0.803	5.67E-04	1.24E-02
NRIP3	nuclear receptor interacting protein 3	0.632	2.64E-03	3.01E-02
NSMCE2	NSE2/MMS21 homolog, SMC5-SMC6 complex SUMO ligase	0.526	6.13E-03	4.88E-02
NSRP1	nuclear speckle splicing regulatory protein 1	0.768	1.15E-03	1.87E-02
NSUN7	NOP2/Sun RNA methyltransferase family member 7	0.929	4.33E-05	2.99E-03
NT5C	5', 3'-nucleotidase, cytosolic	-1.033	2.28E-03	2.75E-02
NT5C2	5'-nucleotidase, cytosolic II	0.555	8.55E-04	1.59E-02
NT5DC1	5'-nucleotidase domain containing 1	0.699	3.64E-04	9.63E-03
NTN4	netrin 4	0.545	1.77E-03	2.42E-02
NTNG1	netrin G1	0.707	3.60E-03	3.58E-02
NTRK1	neurotrophic receptor tyrosine kinase 1	-2.558	6.96E-06	1.30E-03
NUFIP1	NUFIP1, FMR1 interacting protein 1	0.489	4.43E-03	4.02E-02
NUFIP2	NUFIP2, FMR1 interacting protein 2	0.614	1.40E-03	2.12E-02
NUP43	nucleoporin 43	0.677	4.91E-03	4.27E-02
NUP58	nucleoporin 58	0.546	3.74E-03	3.65E-02
NUP133	nucleoporin 133	0.498	4.05E-03	3.81E-02
NUP153	nucleoporin 153	0.48	1.03E-03	1.76E-02
NUP210L	nucleoporin 210 like	1.015	1.84E-05	2.23E-03
NXF3	nuclear RNA export factor 3	0.832	8.34E-04	1.58E-02
OAZ1	ornithine decarboxylase antizyme 1	-0.84	1.64E-03	2.31E-02
OCLN	occludin	0.756	2.19E-03	2.68E-02
OFD1	OFD1, centriole and centriolar satellite protein	0.502	3.54E-03	3.57E-02
OLFML2A	olfactomedin like 2A	-1.702	9.07E-04	1.65E-02
OLFML2B	olfactomedin like 2B	-0.995	5.15E-03	4.39E-02
ORC2	origin recognition complex subunit 2	0.732	2.19E-03	2.68E-02
OSBPL9	oxysterol binding protein like 9	0.63	3.01E-03	3.26E-02
OSER1	oxidative stress responsive serine rich 1	0.515	1.10E-03	1.82E-02
OSR2	odd-skipped related transciption factor 2	-0.986	1.47E-03	2.19E-02
OTUD4	OTU deubiquitinase 4	0.553	3.26E-04	9.27E-03

OVGP1	oviductal glycoprotein 1	0.724	1.74E-04	6.77E-03
OXCT1	3-oxoacid CoA-transferase 1	0.554	5.77E-03	4.68E-02
P2RX4	purinergic receptor P2X 4	-0.766	5.88E-06	1.21E-03
P4HB	prolyl 4-hydroxylase subunit beta	-0.7	4.73E-04	1.12E-02
PABPC1	poly(A) binding protein cytoplasmic 1	0.507	1.20E-03	1.92E-02
PACRGL	PARK2 coregulated like	0.63	5.53E-03	4.59E-02
PALB2	partner and localizer of BRCA2	0.886	7.33E-05	4.04E-03
PAM	peptidylglycine alpha-amidating monooxygenase	0.632	7.05E-04	1.42E-02
PAN2	PAN2 poly(A) specific ribonuclease subunit	0.387	2.70E-03	3.04E-02
PAPD5	poly(A) RNA polymerase D5, non-canonical	0.551	5.18E-03	4.40E-02
PAPOLG	poly(A) polymerase gamma	0.634	3.17E-03	3.34E-02
PARD3	par-3 family cell polarity regulator	0.437	5.61E-03	4.61E-02
PARP3	poly(ADP-ribose) polymerase family member 3	-1.217	4.39E-03	4.01E-02
PARP9	poly(ADP-ribose) polymerase family member 9	0.742	5.63E-03	4.62E-02
PAXBP1	PAX3 and PAX7 binding protein 1	0.7	1.89E-03	2.50E-02
PCBP1	poly(rC) binding protein 1	-0.663	2.68E-03	3.03E-02
PCLO	piccolo presynaptic cytomatrix protein	1.103	4.55E-06	1.07E-03
PCM1	pericentriolar material 1	0.617	3.41E-03	3.48E-02
PCOLCE2	procollagen C-endopeptidase enhancer 2	1.616	4.23E-03	3.93E-02
PCYT2	phosphate cytidylyltransferase 2, ethanolamine	-1.655	2.56E-04	8.25E-03
PDE7A	phosphodiesterase 7A	0.712	4.32E-05	2.99E-03
PDIK1L	PDLIM1 interacting kinase 1 like	0.599	3.64E-03	3.59E-02
PDP1	pyruvate dehyrogenase phosphatase catalytic subunit 1	0.415	4.55E-03	4.08E-02
PDP2	pyruvate dehyrogenase phosphatase catalytic subunit 2	-0.567	2.65E-03	3.01E-02
PDZD8	PDZ domain containing 8	0.565	1.91E-03	2.51E-02
PEX1	peroxisomal biogenesis factor 1	0.507	2.84E-03	3.13E-02
PFDN5	prefoldin subunit 5	0.446	5.44E-03	4.53E-02
PFKFB4	6-phosphofructo-2-kinase/fructose-2,6-biphosphatase 4	-0.967	4.53E-03	4.07E-02
PGM2L1	phosphoglucomutase 2 like 1	0.911	2.19E-04	7.58E-03
PHACTR4	phosphatase and actin regulator 4	0.341	6.28E-03	4.94E-02
PHF3	PHD finger protein 3	0.515	1.65E-03	2.31E-02
PHF7	PHD finger protein 7	0.707	3.35E-04	9.43E-03

PHF20	PHD finger protein 20	0.485	5.53E-04	1.23E-02
PHF21A	PHD finger protein 21A	0.393	1.25E-04	5.50E-03
PHIP	pleckstrin homology domain interacting protein	0.627	3.10E-03	3.30E-02
PI4K2B	phosphatidylinositol 4-kinase type 2 beta	0.736	4.44E-03	4.02E-02
PIAS1	protein inhibitor of activated STAT 1	0.531	3.57E-03	3.57E-02
PIGW	phosphatidylinositol glycan anchor biosynthesis class W	0.753	1.45E-03	2.18E-02
PIK3C3	phosphatidylinositol 3-kinase catalytic subunit type 3	0.633	1.11E-03	1.82E-02
PIKFYVE	phosphoinositide kinase, FYVE-type zinc finger containing	0.518	1.06E-03	1.79E-02
PIWIL2	piwi like RNA-mediated gene silencing 2	0.892	1.97E-04	7.18E-03
PIWIL4	piwi like RNA-mediated gene silencing 4	1.223	1.74E-05	2.22E-03
PKD2	polycystin 2, transient receptor potential cation channel	0.657	3.65E-03	3.59E-02
PKD1L3	polycystin 1 like 3, transient receptor potential channel interacting	0.594	5.09E-03	4.36E-02
PLCE1	phospholipase C epsilon 1	0.469	5.69E-03	4.64E-02
PLCG2	phospholipase C gamma 2	-0.914	2.35E-04	7.90E-03
PLCH1	phospholipase C eta 1	0.9	1.23E-05	1.78E-03
PLEKHA3	pleckstrin homology domain containing A3	0.709	3.00E-04	8.90E-03
PLEKHA5	pleckstrin homology domain containing A5	0.953	3.83E-07	5.62E-04
PLEKHF2	pleckstrin homology and FYVE domain containing 2	0.761	2.61E-03	2.99E-02
PLEKHH2	pleckstrin homology, MyTH4 and FERM domain containing H2	0.624	3.20E-04	9.20E-03
PLIN3	perilipin 3	-1.668	3.92E-04	9.96E-03
PLK4	polo like kinase 4	0.687	3.67E-03	3.61E-02
Pln	phospholamban	1.24	2.77E-03	3.09E-02
Plpp1	phospholipid phosphatase 1	0.669	8.66E-05	4.43E-03
PLPP3	phospholipid phosphatase 3	-1.175	2.74E-06	9.04E-04
PLXDC1	plexin domain containing 1	-1.156	2.53E-03	2.93E-02
PM20D2	peptidase M20 domain containing 2	0.721	5.37E-03	4.49E-02
PMFBP1	polyamine modulated factor 1 binding protein 1	1.314	2.00E-06	7.83E-04
PMS1	PMS1 homolog 1, mismatch repair system component	0.731	6.28E-03	4.94E-02
PMVK	phosphomevalonate kinase	-1.642	5.28E-04	1.20E-02
PNPO	pyridoxamine 5'-phosphate oxidase	-0.844	1.46E-03	2.18E-02
PNPT1	polyribonucleotide nucleotidyltransferase 1	0.848	6.32E-04	1.34E-02
POGZ	pogo transposable element with ZNF domain	0.381	6.46E-04	1.34E-02

POLD3	DNA polymerase delta 3, accessory subunit	0.616	2.46E-03	2.88E-02
POLE2	DNA polymerase epsilon 2, accessory subunit	0.827	3.54E-03	3.57E-02
POLR3B	RNA polymerase III subunit B	0.436	5.03E-03	4.34E-02
POLR3G	RNA polymerase III subunit G	0.971	1.32E-04	5.65E-03
PPAT	phosphoribosyl pyrophosphate amidotransferase	0.643	1.26E-03	1.98E-02
PPEF1	protein phosphatase with EF-hand domain 1	0.793	3.14E-03	3.33E-02
PPID	peptidylprolyl isomerase D	0.58	7.73E-04	1.51E-02
PPIL4	peptidylprolyl isomerase like 4	0.659	5.22E-03	4.43E-02
PPIP5K2	diphosphoinositol pentakisphosphate kinase 2	0.752	3.02E-03	3.26E-02
PPM1K	protein phosphatase, Mg2+/Mn2+ dependent 1K	0.815	3.34E-03	3.43E-02
PPP1R3C	protein phosphatase 1 regulatory subunit 3C	1.174	9.19E-04	1.67E-02
PPP2R5A	protein phosphatase 2 regulatory subunit B'alpha	0.622	2.43E-03	2.86E-02
PPP3R1	protein phosphatase 3 regulatory subunit B, alpha	0.624	1.86E-03	2.49E-02
PPP4R4	protein phosphatase 4 regulatory subunit 4	0.691	1.37E-03	2.08E-02
PPP4R3A	protein phosphatase 4 regulatory subunit 3A	0.506	4.00E-03	3.79E-02
PPT1	palmitoyl-protein thioesterase 1	0.611	6.22E-03	4.91E-02
PRDM4	PR/SET domain 4	0.463	8.29E-04	1.58E-02
PRDM5	PR/SET domain 5	0.559	1.91E-03	2.50E-02
PREX1	phosphatidylinositol-3,4,5-trisphosphate dependent Rac exchange factor 1	-1.136	1.54E-04	6.28E-03
PRIM2	primase (DNA) subunit 2	0.652	1.01E-03	1.74E-02
PRKCQ	protein kinase C theta	0.527	3.44E-03	3.49E-02
PROM1	prominin 1	0.911	6.97E-04	1.40E-02
PRPF3	pre-mRNA processing factor 3	0.54	1.91E-03	2.50E-02
PRPF39	pre-mRNA processing factor 39	0.7	4.28E-03	3.95E-02
PRRC2C	proline rich coiled-coil 2C	0.45	1.58E-03	2.27E-02
PRSS35	protease, serine 35	0.935	1.82E-03	2.47E-02
PSIP1	PC4 and SFRS1 interacting protein 1	1.03	2.18E-05	2.31E-03
PSMA7	proteasome subunit alpha 7	-0.553	5.98E-03	4.80E-02
PSMA8	proteasome subunit alpha 8	1.047	2.32E-04	7.89E-03
PSMB2	proteasome subunit beta 2	-0.488	1.38E-03	2.10E-02
PSMB3	proteasome subunit beta 3	-0.444	1.20E-03	1.92E-02
PSMB4	proteasome subunit beta 4	-0.463	2.00E-03	2.55E-02

PSMB5	proteasome subunit beta 5	-0.553	3.48E-04	9.57E-03
PSMB8	proteasome subunit beta 8	-0.702	2.55E-03	2.94E-02
PTAFR	platelet activating factor receptor	-0.623	1.57E-03	2.27E-02
PTCD3	pentatricopeptide repeat domain 3	0.588	8.07E-04	1.55E-02
PTGES3	prostaglandin E synthase 3	0.715	3.49E-03	3.53E-02
PTPN3	protein tyrosine phosphatase, non-receptor type 3	0.811	6.77E-05	3.86E-03
PTPN12	protein tyrosine phosphatase, non-receptor type 12	0.66	6.24E-03	4.93E-02
PTPRB	protein tyrosine phosphatase, receptor type B	0.678	2.02E-03	2.57E-02
PTPRZ1	protein tyrosine phosphatase, receptor type Z1	1.062	1.74E-06	7.65E-04
PTX3	pentraxin 3	-0.785	1.18E-03	1.90E-02
PUS7	pseudouridylate synthase 7 (putative)	0.777	3.60E-04	9.63E-03
QPCTL	glutaminyl-peptide cyclotransferase like	-1.095	2.10E-03	2.62E-02
QPRT	quinolinate phosphoribosyltransferase	-2.139	2.53E-04	8.25E-03
QRSL1	glutaminyl-tRNA synthase (glutamine-hydrolyzing)-like 1	0.501	1.65E-03	2.31E-02
QSER1	glutamine and serine rich 1	0.679	8.46E-04	1.59E-02
R3HCC1L	R3H domain and coiled-coil containing 1 like	0.666	3.00E-03	3.25E-02
RAB2B	RAB2B, member RAS oncogene family	0.568	3.62E-03	3.58E-02
RAB3IP	RAB3A interacting protein	1.082	3.70E-05	2.79E-03
RACGAP1	Rac GTPase activating protein 1	0.464	4.05E-03	3.81E-02
RAD50	RAD50 double strand break repair protein	0.836	4.53E-04	1.10E-02
RAD9B	RAD9 checkpoint clamp component B	1.343	3.38E-06	9.60E-04
RANBP9	RAN binding protein 9	0.602	1.17E-03	1.89E-02
RANBP17	RAN binding protein 17	0.758	5.64E-04	1.24E-02
RAPH1	Ras association (RalGDS/AF-6) and pleckstrin homology domains 1	0.478	3.04E-03	3.28E-02
RARRES2	retinoic acid receptor responder 2	-1.607	1.63E-03	2.31E-02
RASA2	RAS p21 protein activator 2	0.761	5.17E-04	1.18E-02
RASAL2	RAS protein activator like 2	0.414	5.65E-03	4.63E-02
RASGEF1B	RasGEF domain family member 1B	0.669	2.26E-03	2.73E-02
RASGRF2	Ras protein specific guanine nucleotide releasing factor 2	0.725	6.29E-04	1.33E-02
RASGRP1	RAS guanyl releasing protein 1	0.75	4.55E-04	1.10E-02
RASSF8	Ras association domain family member 8	0.565	2.65E-03	3.01E-02
RB1CC1	RB1 inducible coiled-coil 1	0.569	3.42E-05	2.70E-03

RBBP6	RB binding protein 6, ubiquitin ligase	0.734	3.49E-04	9.57E-03
RBBP8	RB binding protein 8, endonuclease	1.109	3.08E-05	2.60E-03
RBM5	RNA binding motif protein 5	0.452	3.62E-03	3.58E-02
RBM6	RNA binding motif protein 6	0.58	1.03E-03	1.77E-02
RBM25	RNA binding motif protein 25	0.503	6.06E-03	4.84E-02
RBM27	RNA binding motif protein 27	0.452	2.23E-03	2.70E-02
RBM44	RNA binding motif protein 44	1.107	2.07E-05	2.29E-03
RBM46	RNA binding motif protein 46	1.307	6.67E-05	3.86E-03
RBMS2	RNA binding motif single stranded interacting protein 2	0.472	1.25E-03	1.98E-02
RBMX	RNA binding motif protein, X-linked	0.421	6.02E-03	4.82E-02
RBMX2	RNA binding motif protein, X-linked 2	0.442	5.80E-03	4.69E-02
RCOR1	REST corepressor 1	0.524	5.28E-04	1.20E-02
RCOR3	REST corepressor 3	0.517	1.71E-03	2.37E-02
RELN	reelin	0.89	4.12E-04	1.03E-02
REPS1	RALBP1 associated Eps domain containing 1	0.448	2.16E-03	2.67E-02
RETSAT	retinol saturase	-0.571	2.27E-03	2.74E-02
REV1	REV1, DNA directed polymerase	0.326	1.68E-03	2.34E-02
RFC4	replication factor C subunit 4	0.688	5.78E-04	1.25E-02
RFWD2	ring finger and WD repeat domain 2	0.583	5.76E-03	4.68E-02
RFWD3	ring finger and WD repeat domain 3	0.762	3.89E-05	2.86E-03
RGN	regucalcin	-1.595	1.81E-04	6.90E-03
RHBDF2	rhomboid 5 homolog 2	-2.373	5.67E-04	1.24E-02
RHBDL2	rhomboid like 2	0.638	6.36E-03	4.98E-02
RHBG	Rh family B glycoprotein (gene/pseudogene)	-2.024	7.11E-06	1.30E-03
RHOB	ras homolog family member B	-0.687	9.06E-04	1.65E-02
RHOBTB3	Rho related BTB domain containing 3	0.666	1.09E-03	1.80E-02
RIC3	RIC3 acetylcholine receptor chaperone	1.072	4.37E-07	5.70E-04
RICTOR	RPTOR independent companion of MTOR complex 2	0.999	1.53E-04	6.23E-03
RIMKLA	ribosomal modification protein rimK like family member A	-1.275	1.45E-03	2.17E-02
RIMKLB	ribosomal modification protein rimK like family member B	1.013	2.78E-07	5.62E-04
RIOK1	RIO kinase 1	0.496	5.17E-03	4.40E-02
RIOK2	RIO kinase 2	0.719	7.59E-04	1.49E-02

RNF2	ring finger protein 2	0.662	1.63E-03	2.31E-02
RNF17	ring finger protein 17	1.294	3.07E-07	5.62E-04
RNF38	ring finger protein 38	0.449	2.34E-03	2.77E-02
RNF138	ring finger protein 138	0.608	5.63E-03	4.62E-02
RNF146	ring finger protein 146	0.57	3.31E-03	3.42E-02
RNF168	ring finger protein 168	0.642	5.48E-03	4.56E-02
RNF216	ring finger protein 216	0.411	1.95E-03	2.52E-02
RNMT	RNA guanine-7 methyltransferase	0.655	4.14E-03	3.87E-02
RNPC3	RNA binding region (RNP1, RRM) containing 3	0.692	4.48E-03	4.04E-02
ROCK1	Rho associated coiled-coil containing protein kinase 1	0.741	1.46E-03	2.18E-02
RPA2	replication protein A2	0.647	8.97E-04	1.64E-02
RPF2	ribosome production factor 2 homolog	0.654	6.40E-03	5.00E-02
RPGRIP1	retinitis pigmentosa GTPase regulator interacting protein 1	1.138	4.10E-05	2.95E-03
RPL5	ribosomal protein L5	0.731	4.59E-03	4.11E-02
RPL14	ribosomal protein L14	0.394	5.77E-03	4.68E-02
RPL23	ribosomal protein L23	0.71	2.77E-03	3.09E-02
RPS6	ribosomal protein S6	0.629	3.08E-03	3.30E-02
RPS24	ribosomal protein S24	0.653	5.66E-03	4.63E-02
RPS27	ribosomal protein S27	0.624	4.63E-03	4.12E-02
RPS27A	ribosomal protein S27a	0.563	4.83E-03	4.22E-02
RPS6KA6	ribosomal protein S6 kinase A6	0.987	2.60E-04	8.33E-03
RRAGB	Ras related GTP binding B	0.629	4.17E-03	3.89E-02
RRAGD	Ras related GTP binding D	0.635	3.60E-04	9.63E-03
RSBN1	round spermatid basic protein 1	0.617	3.82E-03	3.69E-02
RTTN	rotatin	0.44	9.59E-04	1.70E-02
RUNDC3B	RUN domain containing 3B	1.043	5.75E-05	3.63E-03
RYR2	ryanodine receptor 2	0.812	5.97E-05	3.67E-03
RYR3	ryanodine receptor 3	1.149	5.41E-06	1.20E-03
S100A14	S100 calcium binding protein A14	-0.797	1.01E-03	1.74E-02
S100A16	S100 calcium binding protein A16	-0.907	9.71E-04	1.70E-02
S100PBP	S100P binding protein	0.567	3.65E-03	3.59E-02
SACM1L	SAC1 suppressor of actin mutations 1-like (yeast)	0.548	5.44E-03	4.53E-02

SACS	sacsin molecular chaperone	0.407	1.55E-03	2.25E-02
SAMD15	sterile alpha motif domain containing 15	0.851	6.05E-04	1.30E-02
SAMM50	SAMM50 sorting and assembly machinery component	-0.468	1.05E-03	1.78E-02
SASS6	SAS-6 centriolar assembly protein	0.897	5.31E-04	1.20E-02
SBNO1	strawberry notch homolog 1	0.688	9.70E-04	1.70E-02
SCAF8	SR-related CTD associated factor 8	0.614	6.77E-04	1.37E-02
SCAF11	SR-related CTD associated factor 11	0.582	5.31E-03	4.47E-02
SCAPER	S-phase cyclin A associated protein in the ER	0.83	5.45E-05	3.50E-03
SCARB1	scavenger receptor class B member 1	-1.816	4.96E-05	3.27E-03
SCEL	sciellin	0.964	2.17E-03	2.68E-02
SCIN	scinderin	0.803	1.87E-03	2.49E-02
SCLT1	sodium channel and clathrin linker 1	0.72	3.59E-03	3.58E-02
SCLY	selenocysteine lyase	-1.695	2.11E-03	2.63E-02
SCML1	sex comb on midleg-like 1 (Drosophila)	0.488	4.15E-03	3.87E-02
SCML2	sex comb on midleg-like 2 (Drosophila)	0.831	8.78E-05	4.44E-03
SCN8A	sodium voltage-gated channel alpha subunit 8	0.586	8.62E-04	1.60E-02
SCRN1	secernin 1	0.772	3.36E-06	9.60E-04
SDAD1	SDA1 domain containing 1	0.697	9.39E-04	1.68E-02
SDHAF4	succinate dehydrogenase complex assembly factor 4	-0.558	1.35E-03	2.07E-02
SEC63	SEC63 homolog, protein translocation regulator	0.694	4.01E-03	3.79E-02
SEC31B	SEC31 homolog B, COPII coat complex component	0.448	6.35E-04	1.34E-02
SEC61B	Sec61 translocon beta subunit	-0.442	6.37E-03	4.99E-02
SECISBP2	SECIS binding protein 2	0.498	2.34E-04	7.90E-03
SEH1L	SEH1 like nucleoporin	0.728	4.60E-04	1.10E-02
SEL1L3	SEL1L family member 3	0.817	2.14E-03	2.66E-02
SEMA5B	semaphorin 5B	-2.348	6.35E-05	3.81E-03
SEMA6D	semaphorin 6D	0.745	4.27E-03	3.95E-02
SENP1	SUMO1/sentrin specific peptidase 1	0.628	1.56E-03	2.26E-02
SENP2	SUMO1/sentrin/SMT3 specific peptidase 2	0.499	3.97E-03	3.77E-02
SEPT3	septin 3	0.674	6.49E-04	1.34E-02
SERAC1	serine active site containing 1	0.475	3.31E-03	3.42E-02
SERPING1	serpin family G member 1	-1.102	3.64E-03	3.59E-02

SERPINH1	serpin family H member 1	-1.041	2.79E-03	3.10E-02
SERTAD4	SERTA domain containing 4	0.713	3.86E-03	3.70E-02
SESTD1	SEC14 and spectrin domain containing 1	0.533	1.66E-03	2.32E-02
SETD2	SET domain containing 2	0.552	1.58E-04	6.33E-03
SETD5	SET domain containing 5	0.367	3.18E-03	3.34E-02
SETD9	SET domain containing 9	0.775	2.97E-03	3.22E-02
SETDB1	SET domain bifurcated 1	0.437	5.44E-04	1.22E-02
SETX	senataxin	0.515	1.88E-03	2.49E-02
SFMBT1	Scm-like with four mbt domains 1	0.771	2.03E-05	2.27E-03
SFRP1	secreted frizzled related protein 1	1.407	8.35E-06	1.42E-03
SGCB	sarcoglycan beta	0.607	5.22E-03	4.43E-02
SHCBP1L	SHC binding and spindle associated 1 like	1.21	7.45E-06	1.33E-03
SIAH1	siah E3 ubiquitin protein ligase 1	0.545	3.72E-03	3.64E-02
SIGLEC1	sialic acid binding Ig like lectin 1	-1.322	1.91E-03	2.51E-02
SIGMAR1	sigma non-opioid intracellular receptor 1	-0.673	8.29E-04	1.58E-02
SIN3A	SIN3 transcription regulator family member A	0.319	4.69E-03	4.15E-02
SIRPA	signal regulatory protein alpha	-0.51	6.16E-03	4.88E-02
SIRT1	sirtuin 1	0.804	8.19E-04	1.57E-02
SKA1	spindle and kinetochore associated complex subunit 1	0.773	1.07E-03	1.80E-02
SKA3	spindle and kinetochore associated complex subunit 3	0.695	3.64E-04	9.63E-03
SKAP1	src kinase associated phosphoprotein 1	0.675	1.20E-03	1.92E-02
SLAMF7	SLAM family member 7	0.934	3.75E-03	3.65E-02
SLC12A6	solute carrier family 12 member 6	0.749	6.85E-05	3.87E-03
SLC16A14	solute carrier family 16 member 14	-1.066	9.25E-07	7.08E-04
SLC24A5	solute carrier family 24 member 5	0.726	4.96E-03	4.30E-02
SLC25A31	solute carrier family 25 member 31	1.041	4.31E-05	2.99E-03
SLC25A33	solute carrier family 25 member 33	0.474	2.42E-03	2.85E-02
SLC25A40	solute carrier family 25 member 40	0.762	4.28E-03	3.95E-02
SLC26A5	solute carrier family 26 member 5	1.146	9.94E-05	4.78E-03
SLC2A3	solute carrier family 2 member 3	0.547	1.92E-03	2.51E-02
SLC2A8	solute carrier family 2 member 8	-1.714	4.07E-04	1.02E-02
SLC30A10	solute carrier family 30 member 10	-0.821	1.93E-03	2.51E-02

SLC4A7	solute carrier family 4 member 7	0.819	3.37E-04	9.44E-03
SLC5A11	solute carrier family 5 member 11	-1.42	6.76E-04	1.37E-02
SLC6A8	solute carrier family 6 member 8	-0.994	6.16E-03	4.88E-02
SLC6A15	solute carrier family 6 member 15	1.278	4.12E-06	1.05E-03
SLC7A6	solute carrier family 7 member 6	0.579	2.42E-04	8.04E-03
SLC8A1	solute carrier family 8 member A1	0.694	1.87E-03	2.49E-02
SLF1	SMC5-SMC6 complex localization factor 1	0.806	7.47E-04	1.48E-02
SLIT2	slit guidance ligand 2	1.062	6.48E-05	3.84E-03
SLTM	SAFB like transcription modulator	0.645	1.31E-03	2.03E-02
SMARCAD1	SWI/SNF-related, matrix-associated actin-dependent regulator of chromatin, subfamily a, containing DEAD/H box 1	0.711	2.14E-03	2.66E-02
SMARCC1	SWI/SNF related, matrix associated, actin dependent regulator of chromatin subfamily c member 1	0.542	1.40E-04	5.84E-03
SMC3	structural maintenance of chromosomes 3	0.883	2.73E-04	8.49E-03
SMC4	structural maintenance of chromosomes 4	0.777	3.12E-03	3.31E-02
SMC5	structural maintenance of chromosomes 5	0.707	2.25E-03	2.73E-02
SMC1B	structural maintenance of chromosomes 1B	1.254	6.73E-07	6.25E-04
SMCHD1	structural maintenance of chromosomes flexible hinge domain containing 1	0.662	2.32E-03	2.76E-02
SMG1	SMG1, nonsense mediated mRNA decay associated PI3K related kinase	0.522	1.35E-03	2.07E-02
SMG7	SMG7, nonsense mediated mRNA decay factor	0.343	1.87E-03	2.49E-02
SMG8	SMG8, nonsense mediated mRNA decay factor	0.544	4.38E-03	4.01E-02
SMURF2	SMAD specific E3 ubiquitin protein ligase 2	0.613	8.25E-04	1.58E-02
SNAP91	synaptosome associated protein 91	0.739	1.11E-03	1.83E-02
SNAPC1	small nuclear RNA activating complex polypeptide 1	0.746	1.39E-03	2.10E-02
SNRNP25	small nuclear ribonucleoprotein U11/U12 subunit 25	-0.942	3.11E-04	9.12E-03
SNRNP48	small nuclear ribonucleoprotein U11/U12 subunit 48	0.439	7.15E-04	1.43E-02
SNRPA1	small nuclear ribonucleoprotein polypeptide A'	0.497	5.72E-04	1.24E-02
SNTB1	syntrophin beta 1	-0.808	3.16E-04	9.16E-03
SNW1	SNW domain containing 1	0.617	1.54E-03	2.24E-02
SNX5	sorting nexin 5	0.561	2.55E-03	2.94E-02
SNX16	sorting nexin 16	0.867	4.61E-03	4.11E-02

SNX25	sorting nexin 25	0.696	1.90E-04	7.06E-03
SOBP	sine oculis binding protein homolog	0.699	5.33E-04	1.20E-02
SOCS5	suppressor of cytokine signaling 5	0.718	3.06E-04	9.01E-03
SORL1	sortilin related receptor 1	0.407	3.62E-03	3.58E-02
SOS2	SOS Ras/Rho guanine nucleotide exchange factor 2	0.721	4.27E-04	1.06E-02
SOX5	SRY-box 5	0.672	3.33E-04	9.39E-03
SP4	Sp4 transcription factor	0.507	5.29E-03	4.46E-02
SPAG1	sperm associated antigen 1	0.715	2.84E-04	8.69E-03
SPAST	spastin	0.758	2.70E-04	8.45E-03
SPATA5	spermatogenesis associated 5	0.731	1.91E-04	7.06E-03
SPATA7	spermatogenesis associated 7	0.781	3.41E-04	9.49E-03
SPATA22	spermatogenesis associated 22	1.32	5.77E-06	1.21E-03
SPATS2	spermatogenesis associated serine rich 2	0.351	4.36E-03	3.99E-02
SPEN	spen family transcriptional repressor	0.315	5.54E-03	4.59E-02
SPICE1	spindle and centriole associated protein 1	0.738	3.19E-04	9.19E-03
SREBF2	sterol regulatory element binding transcription factor 2	-1.134	4.52E-03	4.07E-02
SRPK1	SRSF protein kinase 1	0.681	2.30E-04	7.84E-03
SRSF12	serine and arginine rich splicing factor 12	0.983	2.19E-05	2.31E-03
SRXN1	sulfiredoxin 1	-0.806	1.48E-03	2.19E-02
STAB2	stabilin 2	0.548	2.07E-03	2.60E-02
STAG1	stromal antigen 1	0.714	6.14E-04	1.31E-02
STAR	steroidogenic acute regulatory protein	-1.914	3.60E-03	3.58E-02
STARD10	StAR related lipid transfer domain containing 10	-0.703	4.78E-03	4.19E-02
STARD13	StAR related lipid transfer domain containing 13	-0.966	6.58E-04	1.35E-02
STC1	stanniocalcin 1	1.035	2.81E-04	8.61E-03
STIM2	stromal interaction molecule 2	0.571	7.74E-04	1.51E-02
STK17A	serine/threonine kinase 17a	0.702	2.92E-03	3.19E-02
STK38L	serine/threonine kinase 38 like	0.628	5.09E-03	4.36E-02
STXBP5	syntaxin binding protein 5	0.605	1.56E-03	2.26E-02
STXBP5L	syntaxin binding protein 5 like	0.774	6.30E-03	4.95E-02
STYXL1	serine/threonine/tyrosine interacting like 1	0.912	4.64E-03	4.13E-02
Sult1a1	sulfotransferase family 1A, phenol-preferring, member 1	-1.428	2.14E-04	7.41E-03

SUOX	sulfite oxidase	-0.995	2.56E-04	8.25E-03
SUPT16H	SPT16 homolog, facilitates chromatin remodeling subunit	0.556	1.98E-03	2.55E-02
SUPT3H	SPT3 homolog, SAGA and STAGA complex component	0.527	3.88E-03	3.72E-02
SUV39H2	suppressor of variegation 3-9 homolog 2	0.7	2.53E-03	2.93E-02
SV2B	synaptic vesicle glycoprotein 2B	1.332	7.99E-06	1.40E-03
SYCP1	synaptonemal complex protein 1	1.16	1.03E-05	1.58E-03
SYCP2	synaptonemal complex protein 2	1.255	3.28E-05	2.64E-03
SYCP3	synaptonemal complex protein 3	1.301	2.91E-05	2.55E-03
SYCP2L	synaptonemal complex protein 2 like	1.298	7.94E-05	4.28E-03
SYDE2	synapse defective Rho GTPase homolog 2	1.251	3.28E-05	2.64E-03
SYT9	synaptotagmin 9	0.794	1.07E-04	5.04E-03
TADA1	transcriptional adaptor 1	0.644	1.99E-03	2.55E-02
TADA2A	transcriptional adaptor 2A	0.643	5.48E-04	1.22E-02
TAF5	TATA-box binding protein associated factor 5	0.874	3.86E-05	2.86E-03
TAF1A	TATA-box binding protein associated factor, RNA polymerase I subunit A	0.8	1.97E-03	2.55E-02
TAF1B	TATA-box binding protein associated factor, RNA polymerase I subunit B	0.884	5.06E-04	1.17E-02
TAF1D	TATA-box binding protein associated factor, RNA polymerase I subunit D	0.758	3.77E-03	3.66E-02
TAOK3	TAO kinase 3	0.433	4.72E-03	4.17E-02
TAP1	transporter 1, ATP binding cassette subfamily B member	-0.921	7.08E-04	1.42E-02
TAP2	transporter 2, ATP binding cassette subfamily B member	-0.954	6.26E-03	4.93E-02
TARBP1	TAR (HIV-1) RNA binding protein 1	0.844	3.70E-05	2.79E-03
TATDN1	TatD DNase domain containing 1	0.751	4.63E-03	4.12E-02
TBC1D4	TBC1 domain family member 4	-0.373	4.19E-03	3.90E-02
TBC1D5	TBC1 domain family member 5	0.439	8.27E-04	1.58E-02
TBC1D30	TBC1 domain family member 30	0.625	1.07E-03	1.80E-02
TBPL1	TATA-box binding protein like 1	0.635	3.09E-03	3.30E-02
TCERG1	transcription elongation regulator 1	0.624	3.81E-04	9.79E-03
TCF7	transcription factor 7 (T-cell specific, HMG-box)	0.609	2.63E-05	2.39E-03
TCHP	trichoplein keratin filament binding	0.45	2.18E-03	2.68E-02
TDRD1	tudor domain containing 1	1.197	1.02E-05	1.58E-03
TDRD3	tudor domain containing 3	0.619	1.74E-03	2.39E-02
TDRD5	tudor domain containing 5	1.073	4.64E-05	3.11E-03

TDRD6	tudor domain containing 6	1.246	2.25E-06	8.01E-04
TDRD7	tudor domain containing 7	0.645	5.06E-04	1.17E-02
TDRD12	tudor domain containing 12	1.06	9.67E-06	1.57E-03
TDRKH	tudor and KH domain containing	0.507	4.74E-03	4.18E-02
TENM3	teneurin transmembrane protein 3	0.489	2.03E-03	2.57E-02
TERF1	telomeric repeat binding factor 1	0.738	2.22E-03	2.70E-02
TERF2	telomeric repeat binding factor 2	0.421	2.49E-03	2.90E-02
TEX10	testis expressed 10	0.648	2.40E-04	8.03E-03
TEX11	testis expressed 11	0.748	1.67E-04	6.55E-03
TEX12	testis expressed 12	1.329	1.69E-04	6.61E-03
TEX14	testis expressed 14, intercellular bridge forming factor	0.761	4.91E-04	1.15E-02
TEX15	testis expressed 15, meiosis and synapsis associated	0.835	3.71E-04	9.73E-03
TEX30	testis expressed 30	0.844	1.10E-03	1.82E-02
TF	transferrin	-1.202	8.39E-05	4.37E-03
TFAP2C	transcription factor AP-2 gamma	0.739	3.69E-04	9.71E-03
THAP5	THAP domain containing 5	0.774	3.86E-03	3.70E-02
THBS1	thrombospondin 1	1.117	7.98E-05	4.28E-03
THBS2	thrombospondin 2	-1.181	1.17E-03	1.89E-02
THG1L	tRNA-histidine guanylyltransferase 1 like	0.451	4.75E-03	4.19E-02
THNSL2	threonine synthase like 2	-0.622	1.61E-03	2.29E-02
THOC1	THO complex 1	0.804	5.67E-04	1.24E-02
TIAL1	TIA1 cytotoxic granule associated RNA binding protein like 1	0.633	4.60E-03	4.11E-02
TIAM2	T-cell lymphoma invasion and metastasis 2	0.48	2.32E-03	2.76E-02
TIMP2	TIMP metallopeptidase inhibitor 2	-0.785	5.14E-05	3.37E-03
TIMP3	TIMP metallopeptidase inhibitor 3	0.567	2.23E-03	2.70E-02
TIPARP	TCDD inducible poly(ADP-ribose) polymerase	0.79	1.20E-04	5.39E-03
TIPIN	TIMELESS interacting protein	0.669	3.06E-03	3.29E-02
TKTL1	transketolase like 1	0.777	5.30E-04	1.20E-02
TLK1	tousled like kinase 1	0.678	7.40E-04	1.47E-02
TM6SF1	transmembrane 6 superfamily member 1	0.783	3.46E-03	3.51E-02
TM7SF2	transmembrane 7 superfamily member 2	-3.47	9.42E-08	2.77E-04
TMEM57	transmembrane protein 57	0.649	1.73E-05	2.22E-03

TMEM164	transmembrane protein 164	-0.642	1.93E-03	2.51E-02
TMEM256	transmembrane protein 256	-0.521	1.15E-03	1.87E-02
TMEM176B	transmembrane protein 176B	-0.913	4.64E-04	1.11E-02
TMEM63A	transmembrane protein 63A	-0.708	5.70E-03	4.64E-02
TMOD1	tropomodulin 1	0.586	3.43E-03	3.49E-02
TNKS	tankyrase	0.367	1.57E-03	2.27E-02
TNNT1	troponin T1, slow skeletal type	-1.268	1.37E-03	2.08E-02
TOM1L1	target of myb1 like 1 membrane trafficking protein	0.843	2.56E-05	2.36E-03
Tomm5	translocase of outer mitochondrial membrane 5 homolog (yeast)	-0.538	2.28E-03	2.75E-02
TOPAZ1	testis and ovary specific PAZ domain containing 1	1.329	5.71E-06	1.21E-03
TOPBP1	topoisomerase (DNA) II binding protein 1	0.538	5.36E-03	4.48E-02
TP53BP1	tumor protein p53 binding protein 1	0.309	6.05E-03	4.84E-02
TPI1	triosephosphate isomerase 1	-0.649	1.51E-03	2.21E-02
TPR	translocated promoter region, nuclear basket protein	0.568	1.65E-03	2.31E-02
TPST1	tyrosylprotein sulfotransferase 1	0.651	2.20E-03	2.69E-02
TRIM2	tripartite motif containing 2	0.633	6.15E-03	4.88E-02
TRIM33	tripartite motif containing 33	0.543	1.83E-03	2.47E-02
TRIM36	tripartite motif containing 36	0.971	7.79E-05	4.24E-03
TRIP12	thyroid hormone receptor interactor 12	0.474	8.53E-04	1.59E-02
TRMT13	tRNA methyltransferase 13 homolog	0.847	2.31E-03	2.76E-02
TROAP	trophinin associated protein	-1.183	2.43E-03	2.85E-02
TRPC1	transient receptor potential cation channel subfamily C member 1	0.753	2.79E-03	3.10E-02
TSC22D3	TSC22 domain family member 3	-0.675	4.96E-03	4.30E-02
TSGA10	testis specific 10	0.839	4.24E-04	1.05E-02
TSHZ2	teashirt zinc finger homeobox 2	-0.955	4.66E-03	4.13E-02
TSPAN15	tetraspanin 15	-0.898	5.91E-03	4.76E-02
TSPAN17	tetraspanin 17	-1.155	2.75E-04	8.52E-03
TTBK2	tau tubulin kinase 2	0.538	3.49E-04	9.57E-03
TTC3	tetratricopeptide repeat domain 3	0.721	6.47E-05	3.84E-03
TTC12	tetratricopeptide repeat domain 12	0.762	1.21E-04	5.41E-03
TTC17	tetratricopeptide repeat domain 17	0.403	1.99E-03	2.55E-02
TTN	titin	1.086	1.09E-05	1.64E-03

TTYH2	tweety family member 2	-1.99	1.12E-06	7.32E-04
TUBD1	tubulin delta 1	0.834	1.19E-03	1.90E-02
TUBGCP5	tubulin gamma complex associated protein 5	0.672	8.45E-04	1.59E-02
TUFT1	tuftelin 1	0.451	3.82E-03	3.69E-02
TXNDC16	thioredoxin domain containing 16	0.7	3.90E-03	3.72E-02
UACA	uveal autoantigen with coiled-coil domains and ankyrin repeats	0.761	1.52E-03	2.22E-02
UBA6	ubiquitin like modifier activating enzyme 6	0.708	2.97E-03	3.22E-02
UBE2C	ubiquitin conjugating enzyme E2 C	-0.626	3.61E-03	3.58E-02
UBE2J1	ubiquitin conjugating enzyme E2 J1	0.541	2.07E-03	2.60E-02
UBE2R2	ubiquitin conjugating enzyme E2 R2	0.359	4.66E-03	4.13E-02
UBP1	upstream binding protein 1 (LBP-1a)	0.446	8.41E-05	4.37E-03
UBR5	ubiquitin protein ligase E3 component n-recognin 5	0.644	3.78E-05	2.83E-03
UCP2	uncoupling protein 2	-0.902	2.17E-03	2.68E-02
UIMC1	ubiquitin interaction motif containing 1	0.616	6.36E-04	1.34E-02
UNC80	unc-80 homolog, NALCN activator	0.849	6.11E-05	3.73E-03
UNC13B	unc-13 homolog B	0.543	1.38E-05	1.92E-03
UPF2	UPF2 regulator of nonsense transcripts homolog (yeast)	0.775	1.56E-04	6.33E-03
URI1	URI1, prefoldin like chaperone	0.916	8.46E-05	4.37E-03
USH2A	usherin	1.157	1.53E-05	2.04E-03
USHBP1	USH1 protein network component harmonin binding protein 1	-1.457	1.12E-04	5.20E-03
USP3	ubiquitin specific peptidase 3	0.506	2.17E-03	2.68E-02
USP12	ubiquitin specific peptidase 12	0.6	9.73E-04	1.70E-02
USP16	ubiquitin specific peptidase 16	0.751	6.41E-04	1.34E-02
USP24	ubiquitin specific peptidase 24	0.511	2.74E-03	3.06E-02
USP26	ubiquitin specific peptidase 26	1.263	2.21E-05	2.31E-03
USP28	ubiquitin specific peptidase 28	0.58	2.66E-04	8.39E-03
USP33	ubiquitin specific peptidase 33	0.738	5.40E-03	4.51E-02
USP37	ubiquitin specific peptidase 37	0.732	4.52E-04	1.10E-02
USP40	ubiquitin specific peptidase 40	0.445	3.78E-03	3.66E-02
USP54	ubiquitin specific peptidase 54	0.539	3.18E-05	2.64E-03
USPL1	ubiquitin specific peptidase like 1	0.684	4.84E-04	1.14E-02
UTP6	UTP6, small subunit processome component	0.75	1.60E-04	6.38E-03

UTP18	UTP18, small subunit processome component	0.684	1.83E-04	6.90E-03
UTP20	UTP20, small subunit processome component	0.719	3.50E-04	9.58E-03
UTP14A	UTP14A small subunit processome component	0.585	8.78E-04	1.62E-02
VAMP4	vesicle associated membrane protein 4	0.647	5.79E-03	4.69E-02
VAMP8	vesicle associated membrane protein 8	-0.667	1.08E-03	1.80E-02
VEZF1	vascular endothelial zinc finger 1	0.472	5.62E-04	1.24E-02
VGLL3	vestigial like family member 3	-1.371	3.26E-05	2.64E-03
VKORC1	vitamin K epoxide reductase complex subunit 1	-0.857	3.14E-03	3.33E-02
VPS8	VPS8, CORVET complex subunit	0.509	3.07E-03	3.29E-02
VPS29	VPS29, retromer complex component	0.656	3.25E-03	3.39E-02
VPS13D	vacuolar protein sorting 13 homolog D	0.349	3.84E-03	3.70E-02
VPS26A	VPS26, retromer complex component A	0.702	2.26E-03	2.73E-02
VWA3B	von Willebrand factor A domain containing 3B	0.811	1.35E-04	5.70E-03
VWA5A	von Willebrand factor A domain containing 5A	0.615	9.52E-04	1.69E-02
WBP4	WW domain binding protein 4	0.809	8.91E-04	1.64E-02
WDHD1	WD repeat and HMG-box DNA binding protein 1	1.075	1.03E-05	1.58E-03
WDR17	WD repeat domain 17	0.681	1.35E-04	5.70E-03
WDR36	WD repeat domain 36	0.682	5.59E-04	1.23E-02
WDR43	WD repeat domain 43	0.706	4.03E-04	1.02E-02
WDR47	WD repeat domain 47	0.618	1.63E-03	2.31E-02
WDR48	WD repeat domain 48	0.567	9.26E-04	1.67E-02
WDR66	WD repeat domain 66	0.951	7.22E-06	1.30E-03
WDR75	WD repeat domain 75	0.784	3.53E-04	9.58E-03
WRN	Werner syndrome RecQ like helicase	0.667	4.43E-03	4.02E-02
WSB1	WD repeat and SOCS box containing 1	0.763	1.42E-03	2.14E-02
WTAP	Wilms tumor 1 associated protein	0.545	1.53E-03	2.24E-02
WWP1	WW domain containing E3 ubiquitin protein ligase 1	0.679	3.46E-04	9.57E-03
XPO5	exportin 5	0.363	3.86E-03	3.70E-02
XXYLT1	xyloside xylosyltransferase 1	-1.083	3.04E-03	3.28E-02
YEATS2	YEATS domain containing 2	0.488	1.97E-04	7.18E-03
YLPM1	YLP motif containing 1	0.443	2.32E-03	2.76E-02
YTHDC1	YTH domain containing 1	0.616	3.42E-03	3.49E-02

YY1	YY1 transcription factor	0.552	4.66E-04	1.11E-02
ZBTB11	zinc finger and BTB domain containing 11	0.677	7.34E-04	1.46E-02
ZBTB20	zinc finger and BTB domain containing 20	-1.427	1.50E-03	2.21E-02
ZBTB26	zinc finger and BTB domain containing 26	0.586	5.00E-03	4.32E-02
ZBTB33	zinc finger and BTB domain containing 33	0.74	5.35E-03	4.48E-02
ZBTB43	zinc finger and BTB domain containing 43	0.523	1.00E-03	1.74E-02
ZBTB44	zinc finger and BTB domain containing 44	0.911	7.03E-05	3.93E-03
ZBTB8A	zinc finger and BTB domain containing 8A	0.687	2.72E-03	3.05E-02
ZC3H8	zinc finger CCCH-type containing 8	0.891	5.25E-05	3.42E-03
ZC3H14	zinc finger CCCH-type containing 14	0.508	8.70E-05	4.43E-03
ZC3H11A	zinc finger CCCH-type containing 11A	0.581	1.60E-03	2.29E-02
ZCCHC4	zinc finger CCHC-type containing 4	0.847	3.45E-05	2.70E-03
ZCCHC8	zinc finger CCHC-type containing 8	0.368	3.77E-03	3.66E-02
ZCWPW1	zinc finger CW-type and PWWP domain containing 1	0.83	1.04E-04	4.99E-03
ZCWPW2	zinc finger CW-type and PWWP domain containing 2	0.803	4.09E-03	3.84E-02
ZDBF2	zinc finger DBF-type containing 2	1.209	3.23E-04	9.24E-03
ZFAND4	zinc finger AN1-type containing 4	0.573	2.11E-03	2.64E-02
ZFP1	ZFP1 zinc finger protein	0.809	9.41E-05	4.61E-03
ZFP2	ZFP2 zinc finger protein	0.938	4.10E-04	1.02E-02
ZFYVE9	zinc finger FYVE-type containing 9	0.719	2.78E-05	2.50E-03
ZKSCAN4	zinc finger with KRAB and SCAN domains 4	0.639	1.35E-04	5.70E-03
ZKSCAN7	zinc finger with KRAB and SCAN domains 7	0.75	6.63E-05	3.86E-03
ZMYM5	zinc finger MYM-type containing 5	0.699	4.40E-03	4.02E-02
ZMYND15	zinc finger MYND-type containing 15	-1.51	1.14E-03	1.86E-02
ZNF2	zinc finger protein 2	0.594	5.18E-03	4.40E-02
ZNF10	zinc finger protein 10	0.734	3.72E-03	3.64E-02
ZNF32	zinc finger protein 32	0.597	2.94E-03	3.20E-02
ZNF35	zinc finger protein 35	0.517	6.06E-04	1.30E-02
ZNF106	zinc finger protein 106	0.637	6.76E-04	1.37E-02
ZNF131	zinc finger protein 131	0.771	4.56E-04	1.10E-02
ZNF180	zinc finger protein 180	0.763	3.79E-04	9.79E-03
ZNF181	zinc finger protein 181	0.631	5.88E-03	4.74E-02

ZNF182	zinc finger protein 182	0.765	1.57E-03	2.27E-02
ZNF184	zinc finger protein 184	0.716	9.95E-04	1.73E-02
ZNF197	zinc finger protein 197	0.676	1.28E-03	2.00E-02
ZNF200	zinc finger protein 200	0.92	5.66E-05	3.59E-03
ZNF211	zinc finger protein 211	0.656	2.92E-03	3.19E-02
ZNF239	zinc finger protein 239	0.68	2.34E-04	7.90E-03
ZNF256	zinc finger protein 256	0.762	2.14E-04	7.41E-03
ZNF263	zinc finger protein 263	0.429	1.99E-03	2.55E-02
ZNF281	zinc finger protein 281	0.413	1.88E-03	2.49E-02
ZNF300	zinc finger protein 300	0.866	5.55E-04	1.23E-02
ZNF317	zinc finger protein 317	0.376	5.22E-03	4.43E-02
ZNF318	zinc finger protein 318	0.733	1.39E-05	1.92E-03
ZNF432	zinc finger protein 432	0.775	8.42E-04	1.59E-02
ZNF445	zinc finger protein 445	0.416	4.76E-03	4.19E-02
ZNF454	zinc finger protein 454	0.683	1.23E-03	1.95E-02
ZNF502	zinc finger protein 502	0.747	8.97E-04	1.64E-02
ZNF507	zinc finger protein 507	0.56	1.00E-03	1.74E-02
ZNF512	zinc finger protein 512	0.488	5.41E-03	4.52E-02
ZNF527	zinc finger protein 527	0.674	4.05E-03	3.81E-02
ZNF555	zinc finger protein 555	0.648	3.42E-03	3.49E-02
ZNF565	zinc finger protein 565	0.63	5.58E-03	4.61E-02
ZNF566	zinc finger protein 566	0.823	3.83E-04	9.79E-03
ZNF596	zinc finger protein 596	0.663	5.32E-03	4.48E-02
ZNF605	zinc finger protein 605	0.884	4.90E-04	1.15E-02
ZNF614	zinc finger protein 614	0.629	4.92E-03	4.27E-02
ZNF638	zinc finger protein 638	0.71	3.07E-03	3.29E-02
ZNF639	zinc finger protein 639	0.748	2.34E-03	2.78E-02
ZNF644	zinc finger protein 644	0.76	4.76E-03	4.19E-02
ZNF654	zinc finger protein 654	0.629	5.83E-03	4.71E-02
ZNF667	zinc finger protein 667	0.775	7.86E-04	1.53E-02
ZNF791	zinc finger protein 791	0.668	3.68E-03	3.61E-02
ZNF879	zinc finger protein 879	0.774	5.97E-03	4.79E-02

ZNF280B	zinc finger protein 280B	0.548	3.12E-03	3.31E-02
ZNF280C	zinc finger protein 280C	0.997	6.30E-05	3.80E-03
ZNF286A	zinc finger protein 286A	0.934	1.55E-06	7.65E-04
ZNF354B	zinc finger protein 354B	0.952	2.29E-04	7.83E-03
ZNF354C	zinc finger protein 354C	0.812	2.55E-03	2.94E-02
ZNHIT6	zinc finger HIT-type containing 6	0.75	1.51E-03	2.21E-02
ZRANB1	zinc finger RANBP2-type containing 1	0.543	2.24E-03	2.72E-02
Zrsr1	zinc finger (CCCH type), RNA binding motif and serine/arginine rich 1	0.688	2.53E-03	2.93E-02
ZRSR2	zinc finger CCCH-type, RNA binding motif and serine/arginine rich 2	0.484	5.67E-03	4.63E-02
ZSCAN12	zinc finger and SCAN domain containing 12	0.902	9.42E-05	4.61E-03
ZSCAN16	zinc finger and SCAN domain containing 16	0.629	3.23E-03	3.38E-02
ZSCAN31	zinc finger and SCAN domain containing 31	0.649	1.28E-03	2.00E-02
ZSCAN5B	zinc finger and SCAN domain containing 5B	0.936	1.36E-03	2.08E-02
ZSWIM5	zinc finger SWIM-type containing 5	0.686	2.67E-03	3.03E-02
ZSWIM6	zinc finger SWIM-type containing 6	0.427	1.88E-03	2.49E-02
ZYG11A	zyg-11 family member A, cell cycle regulator	1.07	1.82E-05	2.23E-03

Supplementary Table 3: Gametogenesis genes differentially expressed in the testes tissue of the Holstein-Friesian dairy bulls feed on a low plane of nutrition in comparison to the high plane of nutrition and slaughtered at 18 weeks of age

Genes	Predicted affect	Expr Log Ratio
SIAH1	Increased	0.545
RNF2	Increased	0.662
MCM9	Increased	0.49
CTCFL	Increased	0.937
NUP153	Increased	0.48
COIL	Increased	0.616
FSHR	Increased	1.021
AGFG1	Increased	0.503
DDX25	Increased	0.781
MCM8	Increased	0.8
KDM3A	Increased	0.615
FADS2	Decreased	-1.09
TSC22D3	Decreased	-0.675
ADAD1	Affected	1.456
NUP210L	Affected	1.015
TSGA10	Affected	0.839
ADAMTS2	Affected	-0.951
SFMBT1	Affected	0.771
TDRKH	Affected	0.507
RGN	Affected	-1.595
ZMYND15	Affected	-1.51
TEX14	Affected	0.761
HORMAD1	Affected	1.121
TDRD1	Affected	1.197
ICA1L	Affected	0.913
TOPAZ1	Affected	1.329
ASZ1	Affected	0.975
CCT6B	Affected	0.772
TDRD5	Affected	1.073
MORC1	Affected	1.425
SYCP2	Affected	1.255
TEX15	Affected	0.835
TEX11	Affected	0.748

Supplementary Table 4 Effect of a high compared to a low plane of nutrition on paired testes weight and on morphological properties including seminiferous tubule diameter and stage of spermatogenesis

	HIGH	LOW	SEM	Significance
Paired testes weight (g)	55.4	31.4	2.91	***
Seminiferous Tubule Diameter (µm)	85.4	72.5	1.76	***
% Gonocyte and Prespermatogonia	31.5	57	1.66	***
% Spermatogonia	68.5	43	1.66	***
No. of Sertoli cells	28	24	0.65	*
Volume Density of Sertoli cells	9.4	8.4	0.35	***

***P<0.001, *P<0.05, SEM = standard error of the mean.
